# Hitherto Unknown Solvent and Anion Pairs in Solvation Structures Reveal New Insights into High‐Performance Lithium‐Ion Batteries

**DOI:** 10.1002/advs.202202405

**Published:** 2022-08-17

**Authors:** Wandi Wahyudi, Xianrong Guo, Viko Ladelta, Leonidas Tsetseris, Mohamad I. Nugraha, Yuanbao Lin, Vincent Tung, Nikos Hadjichristidis, Qian Li, Kang Xu, Jun Ming, Thomas D. Anthopoulos

**Affiliations:** ^1^ KAUST Solar Center King Abdullah University of Science and Technology (KAUST) Thuwal 23955–6900 Saudi Arabia; ^2^ Core Labs King Abdullah University of Science and Technology (KAUST) Thuwal 23955–6900 Saudi Arabia; ^3^ KAUST Catalysis Center King Abdullah University of Science and Technology (KAUST) Thuwal 23955–6900 Saudi Arabia; ^4^ Department of Physics National Technical University of Athens Athens GR‐15780 Greece; ^5^ Research Center for Advanced Materials National Research and Innovation Agency (BRIN) South Tangerang Banten 15314 Indonesia; ^6^ State Key Laboratory of Rare Earth Resource Utilization Changchun Institute of Applied Chemistry Chinese Academy of Sciences Changchun 130022 People's Republic of China; ^7^ Battery Science Branch US Army Research Laboratory Adelphi Maryland 20783 USA

**Keywords:** electrolytes, lithium‐ion batteries, nuclear magnetic resonance (NMR) spectroscopy, solvation structure, solvent and ion pairs

## Abstract

Solvent‐solvent and solvent‐anion pairings in battery electrolytes have been identified for the first time by nuclear magnetic resonance spectroscopy. These hitherto unknown interactions are enabled by the hydrogen bonding induced by the strong Lewis acid Li^+^, and exist between the electron‐deficient hydrogen (*δ* ^+^H) present in the solvent molecules and either other solvent molecules or negatively‐charged anions. Complementary with the well‐established strong but short‐ranged Coulombic interactions between cation and solvent molecules, such weaker but longer‐ranged hydrogen‐bonding casts the formation of an extended liquid structure in electrolytes that is influenced by their components (solvents, additives, salts, and concentration), which in turn dictates the ion transport within bulk electrolytes and across the electrolyte‐electrode interfaces. The discovery of this new inter‐component force completes the picture of how electrolyte components interact and arrange themselves, sets the foundation to design better electrolytes on the fundamental level, and probes battery performances.

## Graphical abstract

Hitherto unknown interactions of solvent–solvent and solvent‐anion pairs between the lithium‐ion (i.e., Li^+^) solvation structures are unveiled for the first time by nuclear magnetic resonance spectroscopy in the battery electrolytes. It has been found that these pairs bridge originally discrete solvation sheaths and lead to the formation of a conjugated solvation network in the electrolytes, which are strongly dependent on the electrolyte components (solvents, additives, salts, and concentration). These findings are significantly important for designing electrolytes on the fundamental level and probing battery performances at a molecular scale.

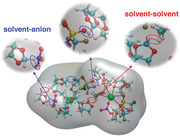

## 1 Introduction

The solvation of ions by solvent molecules underpins a broad range of chemical, physical, and biological processes essential to our world, as exemplified by its integral applications in key industries such as oil refining, water desalination, pharmaceutical, and electrochemical energy storage (e.g., supercapacitors and batteries).^[^ ^1^ ^]^ In the latter, an essential component is non‐aqueous liquid electrolytes and the interphase chemistries they generate.^[^ ^2^ ^]^ Hence, how to tailor and manipulate ion transport in the bulk electrolyte and across electrode‐electrolyte interphases constitute the key knowledge for rational design and engineering of better energy storage devices, especially when more aggressive anode chemistries such as silicon, lithium, potassium, or sodium metal are being pursued.^[^ ^3^ ^]^ In classical ionics, as represented by the Debye–Hückel theory, the electrolyte structure is dominated by the Coulombic interactions among oppositely‐charged ions and polar solvent molecules,^[^ ^4^ ^]^ which are strong but rapidly decay with the square of distance, rendering the solvation sheath structures a local arrangement on the length‐scale less than 0.1 nm between solvent molecules and ions. More recent studies on non‐aqueous electrolytes further established that Li^+^‐solvation sheath structure directly affects the interphasial chemistries, thus linking the cation‐solvent interaction to the interfacial behaviors.^[^ ^5^ ^]^ However, a precise understanding of the interactions among the electrolyte components and the ensuing remains incomplete, rendering the efforts to design better electrolytes unrealistic on a fundamental level.

Understanding of dynamic microstructures in battery electrolytes has been attempted with Raman,^[^ ^6^ ^]^ Fourier‐transform infrared (FTIR),^[^ ^7^ ^]^ and nuclear magnetic resonance (NMR) techniques.^[^ ^8^ ^]^ Unfortunately, the spectroscopic techniques of high temporal resolutions (i.e., Raman and FTIR) are insensitive to structural changes in the electrolyte induced by weak interactions, while extracting accurate information from these experiments encounters inevitable artifacts caused by data deconvolution, leading to the study of cation and anion pairings in electrolytes remains challenging. NMR spectroscopy enables sensitive detection of structural variations at the atomic level, however, all studies so far have overwhelmingly focused on the strong interactions between cation and solvent molecules because of the strong Coulombic field strength of the former, with the role of anions often left in oblivion, and solvent‐solvent interactions not even being aware of. Thus, although the intermolecular interactions via hydrogen bonding have been known to outact van der Waals interaction,^[^ ^9^ ^]^ similar interactions have never been explored in non‐aqueous electrolytes. Among the limited NMR studies on such electrolytes, Xu and coworkers reported that the ^17^O NMR spectra broaden and disappear as the electrolyte concentration increases,^[^ ^8^, ^10^ ^]^ which arises from the strong Coulombic interaction between the cation (Li^+^) and the carbonyl‐O in the carbonate molecule, and indicates that the quadrupolar nature of ^17^O nucleus makes the study of electrolyte microstructures particularly challenging.^[^ ^6^, ^7^, ^11^ ^]^ Fortunately, hydrogen bondings, e.g., CH···O and NH···O, have been rigorously studied in biological and chemical systems,^[^ ^12^ ^]^ of which the achieved state‐of‐the‐art of the study merits translation into battery systems despite the complexity of the electrolytes caused by various electronegative atoms and functional groups in the structure of cations.

Here, we probe the interactions among the electrolyte components that are induced by weak forces like hydrogen bonds using a comprehensive NMR analysis. For the first time, we discover solvent‐solvent and solvent‐anion pairs that present in the battery electrolytes, weak but long‐ranged interactions that shape the extended liquid structure. The formation of the pairs is confirmed in various lithium battery electrolyte systems. Moreover, the properties of the pairs are strongly dependent on the electrolyte components that encompass solvents, additives, salts, and their concentration. These findings complete the relevant solvation structure of electrolytes and advance our understanding of how electrolyte components interact with each other. Furthermore, we show that the solvent and anion pairs enable us to unravel the relationship between electrolyte microstructure and battery performances, giving a better probe than the commonly known cation solvation and cation‐anion pairings. More importantly, this work paves the way to a new guideline for designing better electrolytes on the fundamental level, particularly by promoting solvent‐solvent and solvent‐anion interactions.

## 2 Results and Discussion

**Figure 1:**
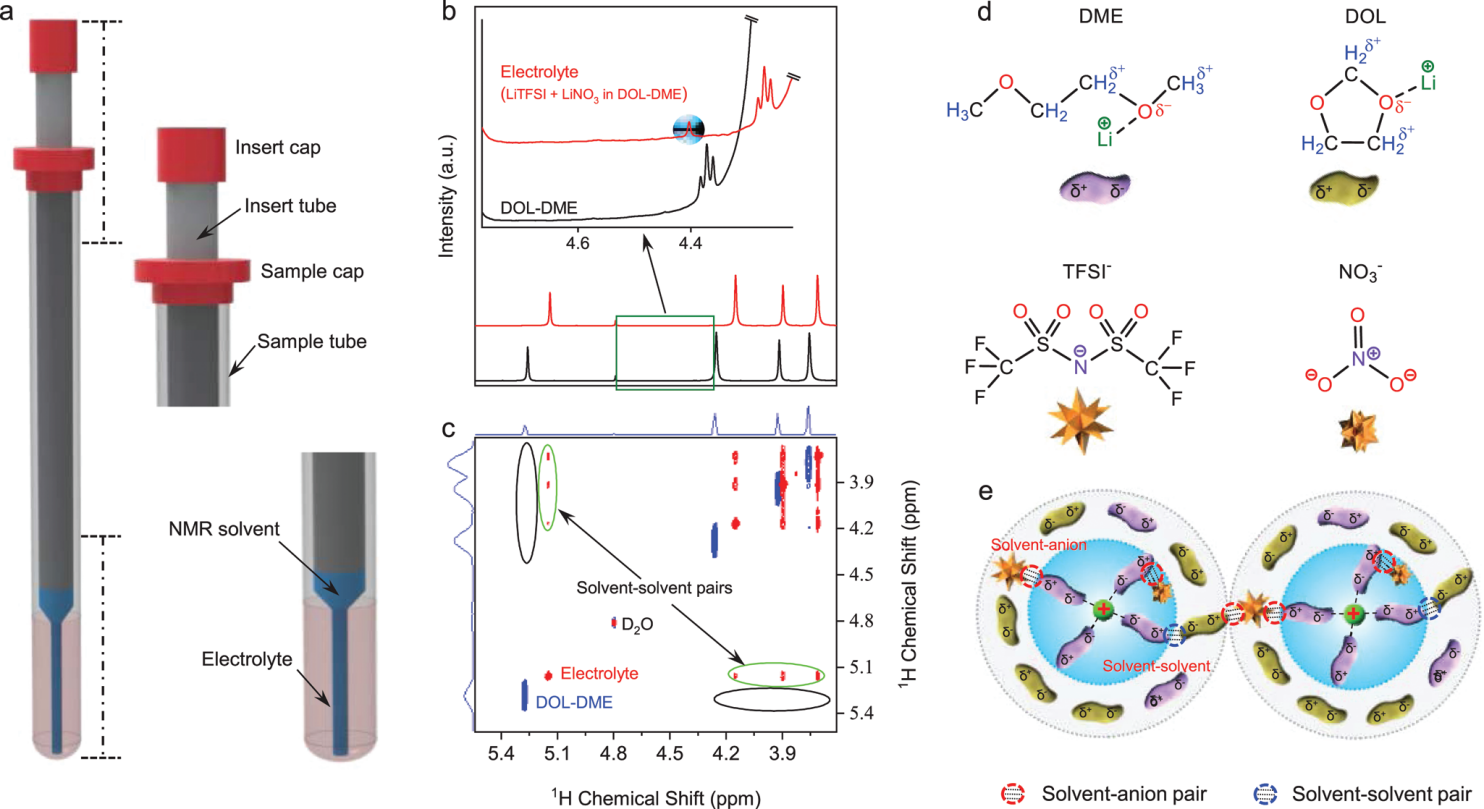
Finding solvent and anion pairs in the electrolyte. a) The electrolyte was added into the NMR tube and then a D_2_O‐filled insert was placed inside the NMR tube. b) ^1^H NMR spectra of neat DOL‐DME and electrolyte showing (inset) a new peak at 4.41 ppm in the electrolyte that corresponds to solvent‐anion pair. c) COSY spectra showing solvent‐solvent pairs (new spectra inside the green circles) in the electrolyte, while the pairs are not observed in the neat DOL‐DME (black circles). d,e) Schematic formation of electron‐deficient hydrogen in the solvents and the pairs upon Li^+^ solvation process.

A coaxial internal insert that contains a standard NMR solvent (i.e., deuterium oxide, D_2_O) was inserted into the NMR tube to enable analysis while preserving the pristine microstructure of the electrolyte (**Figure** **1**a), thus closely simulating the electrolyte environment in the battery. An experimental ether‐based electrolyte that is often used for the emerging lithium/sulfur chemistries,^[^ ^13^ ^]^ i.e., 1.0 M LiN(CF_3_)_2_(SO_2_)_2_ (LiTFSI) and 0.4 M LiNO_3_ dissolved in 1,3‐dioxolane (DOL) and 1,2‐dimethoxyethane (DME) was used as the initial sample. We have found that the ^1^H NMR spectra shift up‐field with respect to the neat DOL‐DME mixture (Figure 1b), confirming that the nuclei are shielded by denser electron clouds and the change in the solvent environment upon Li^+^ solvation process (Figure S1 and S2; Table S1, Supporting Information).^[^ ^14^ ^]^ Meanwhile, a new peak appears at 4.41 ppm, corresponding to the formation of solvent‐anion pair (inset Figure 1b and Figure S3, Supporting Information) that is absent in the neat solvent mixture. The pair was previously believed to be non‐existent due to the weak Coulombic attractions between anion and solvent molecules,^[^ ^5^ ^]^ but the following in‐depth investigation proves its existence and key roles in the batteries. Furthermore, the 2D ^1^H‐^1^H correlation spectroscopy (COSY) in Figure 1c reveals an additional solvent‐solvent interaction (inside the green circles) that should arise from the hydrogen bonding between the neighboring solvent molecules,^[^ ^15^ ^]^ which is again absent in the neat DOL‐DME mixture. Such solvent‐solvent pair presence is hitherto never known in electrolytes, and its formation and potential effects on battery performances merit rigorous examination. The discovery of these solvent and anion pairings benefits from the isolation of the electrolyte by using the internal insert, so that there is no interaction between the electrolyte and the NMR solvent. The control experiment using the standard liquid NMR procedure, i.e., the electrolyte is diluted in the NMR solvents, leads to a significant change in the electrolyte microstructure, and then the pairs are invisible (Figure S4, Supporting Information), suggesting the importance of preserving the nature of electrolytes to obtain detailed information from the microstructure.

The pair formation begins with apparent polarization of electron clouds in the ^1^H‐nuclei of solvents towards the oxygen when the oxygen directly interacts with Li^+^ (Figure 1d), leaving the former partially electron‐deficient (*δ* ^+^H) while rendering the latter electron‐rich (*δ* ^–^O). The strong Lewis acid center Li^+^ thus induces a shift of electron density from hydrogen to oxygen, and enables the *δ* ^+^H to interact with other solvent molecules in the vicinity as well as with the electron‐rich anions of TFSI^–^ and NO_3_^–^, where N, F, and O carries most of the formal charge distributed across the entire anion structure.^[^ ^7^, ^9^ ^]^ Figure 1e schematically shows the solvent‐anion and solvent‐solvent pairs among the neighboring solvation sheaths, incorporating the long‐ranged interactions of *δ* ^+^H‐solvent and *δ* ^+^H‐anion. Self‐diffusion analysis reveals that the solvent‐anion pair diffuses much slower than the neat DOL and DME molecules (Figure S5, Supporting Information), a strong indication that the pair is integral to the solvation sheath. Thus, the pairs are dependent on the electrolyte composition as well as the solvation structure judged by the formula of Li^+^[solvent]_x_[additive]_y_[anion]*_z_*, where *x*, *y*, and *z* are calculated from the molar concentration of electrolytes.^[^ ^16^ ^]^ For example, in the 1.0 M LiTFSI, 0.4 M LiNO_3_ in DOL‐DME electrolyte, the solvents predominantly appear as free molecules and exist in the secondary solvation sheath (Figure 1e), derived from Li^+^[DME]*_3.4_* [DOL]*_5.1_* [NO_3_^–^]*_0.28_* [TFSI^–^]*_0.72_* (Table S2, Supporting Information). Such an excessive population of free solvents interferes with the minor existence of the pairs and then results in a weak intensity of the pairs. In stark contrast, the intensity of the pairs becomes notably stronger with alteration of the electrolyte composition (e.g., solvents, salts, additives, etc.), giving immense effects on the solvation structure and battery performances as described in the following.

**Figure 2:**
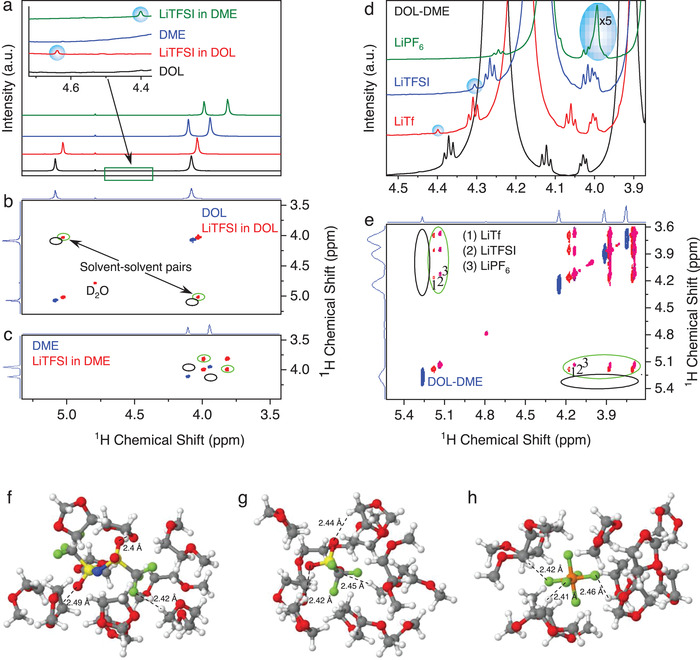
Solvent and anion dependencies of the pairs. Solvent dependence. a) ^1^H NMR spectra showing solvent‐anion pairs in the DOL and DME‐based electrolytes. b,c,) COSY spectra showing solvent–solvent pairs (green circles) in the electrolytes but no pairs detected in the neat solvent (black circles). Anion dependence. d) ^1^H NMR spectra showing solvent‐anion pairs with different chemical shift and intensity in the electrolytes incorporating 1.0 M of LiTf (4.40 ppm), LiTFSI (4.31 ppm), and LiPF_6_ (3.99 ppm) salts. e) COSY spectra of the electrolytes showing solvent‐solvent pairs (green circles) dependence on the type of anion. DFT calculations results of solvent‐anion clusters comprising 4 DOL and 6 DME molecules upon the insertion of f) TFSI^–^, g) Tf^–^, and h) PF_6_^–^ anions (C: gray, H: white, O: red, F: green, S: yellow, N: blue spheres).

The investigation of the solvent and anion pairs is confirmed by their dependencies on the type of solvents and anions (**Figure** **2**), which also reconfirms that the solvent and anion pairings are consistent and intrinsic to the electrolytes, instead of arising from any impurity or artifacts. First, we observe that the ^1^H and ^17^O NMR spectra of the solvents consistently change with the type of solvents and anions in the electrolytes (Figure S6, Supporting Information), confirming the change in the solvent environment upon Li^+^ solvation. Furthermore, the solvent‐anion pair in the DME‐LiTFSI electrolyte is more shielded (4.40 ppm, Figure 2a) than its counterpart in the DOL‐LiTFSI electrolyte (4.64 ppm), reflecting a stronger interaction of TFSI^‐^ with DME due to more electropositive of *δ* ^+^H in the DME, while COSY spectra confirm the presence of the solvent‐solvent pair in both electrolytes (Figure 2b,c). The solvent‐anion pair also depends on the type of anions (Figure 2d), such as LiCF_3_SO_3_ (LiTf; 4.40 ppm; integration ratio to D_2_O is 0.0206), LiTFSI (4.31 ppm; integration 0.0677), and LiPF_6_ (3.99 ppm; integration 0.9349). The magnitude of the solvent‐anion interaction follows the anion order of PF_6_^–^ > TFSI^–^ > Tf^–^, which may be affected by the Lewis basicity of the anions. The self‐diffusion analysis further corroborates the results, the solvent‐anion pair in the LiPF_6_ DOL‐DME electrolyte diffuses much slower than that in the LiTf and LiTFSI systems, indicating the formation of much larger solvation complexes (Figure S7, Supporting Information). Furthermore, the magnitude of the solvent‐solvent pairs also follows the order for solvent‐anion pairs (COSY spectra in Figure 2e), highlighting the key role that anions play in dictating the electrolyte microstructures.

Density functional theory (DFT) calculations (Figure 2f–h) yield an energy gain of 0.83, 0.76, and 0.61 eV upon the insertion of PF_6_^–^, Tf^–^, and TFSI^–^ anions, respectively, in a cluster comprising each anion with six DOL and four DME molecules. The average minimum value for the distances between the electronegative TFSI^–^ and proximate H atoms (within a range of 2.30–2.80 Å) is 2.62 Å (2.40 Å), while the minimum distances involving Tf^–^ and PF_6_^–^ anions are 2.59 Å (2.32 Å) and 2.55 Å (2.33 Å), respectively. All these values are below the pertinent sums of van der Waals radii, once again indicating the formation of rigid hydrogen bonds that can, in turn, give rise to potential new NMR peaks. Interestingly, the electrolyte incorporating LiPF_6_ in DOL‐DME shows the strongest pairs, but after one day, the electrolyte formed solid solvation complexes with properties unlike the neat salt and solvents (Figure S8, Supporting Information). The observed large solvation complexes in LiPF_6_ DOL‐DME electrolyte are consistent with the strong solvent‐anion pair induced by LiPF_6_ (Figure 2d). Our findings thus reveal the crucial nature of the local structure of the solvents and anions in the electrolytes. Considering that the hydrogen bonding, although weaker than Coulombic forces in short‐range (<0.1 nm), is a long‐range interaction, the above *δ* ^+^H‐solvent and *δ* ^+^H‐anion interactions should extend throughout the entire electrolyte bulk solution, and likewise cast strong influence over how the electrolyte‐electrode interface should be structured.

**Figure 3:**
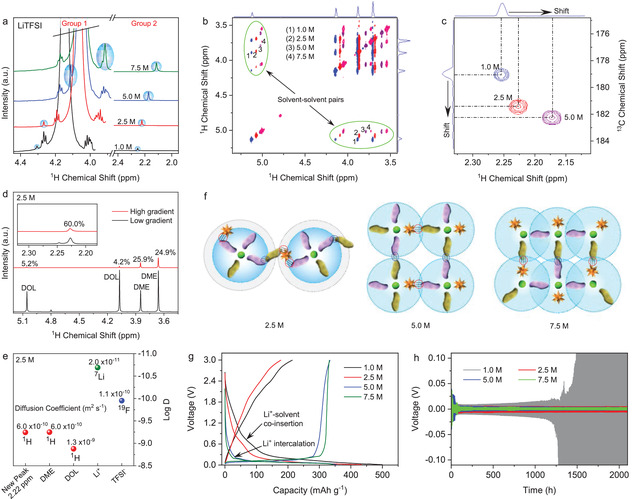
Properties of the solvent and anion pairs. a) ^1^H NMR, b) COSY, and c) heteronuclear multiple bond correlation (HMBC) spectra of the pairs in different concentration of ether‐based electrolytes. d) ^1^H self‐diffusion spectra of 2.5 M electrolyte and e) DOSY spectra of the solvent‐anion pair at 2.228 ppm, DOL, DME, TFSI^–^, and Li^+^. f) Schematic formation of conjugated solvation networks promoted by the pairs. g) Charge–discharge curve of Li vs. graphite cells (current density 300 mAh cm^–2^) and h) Li^+^ stripping‐platting curve of Li vs. Li symmetric cells (current density 500 mAh cm^–2^), showing improved performances when the pairs get stronger. The reference was 0.1 M LiPF_6_ in 1 vol.% H_2_O + 10 vol.% C_2_H_5_OH + 98 vol.% D_2_O solution in the NMR insert.

In addition to the new peak around 4.4–3.9 ppm (group 1), we have found more evidence of the new solvent‐anion pair in the region of 2.0–2.3 ppm ^1^H NMR spectra (Group 2, **Figure** **3**a). The ^1^H‐nuclei of the pairs from both groups consistently turn to be more shielded with increasing salt concentration, which is a natural result of the higher population of negatively‐charged anions in the electrolyte and the corresponding increase in *δ* ^+^H‐anion as well as *δ* ^+^H‐solvent pair formations (Figure 3b). The solvent‐anion pairs in Group 2 associate with newly appear ketone groups in the electrolytes (i.e., R₂C = O) at 178.0–184.0 ppm in the ^13^C NMR (Figure 3c), giving more evidence of solvent and anion interactions in the solvation sheaths. Fourier transform infrared (FTIR) analysis further confirms the finding, where the C–O vibration band of DME at 1105 cm^–1^ disappears while a new C–O vibration band at 1136 cm^–1^ appears upon increasing the electrolyte concentration (Figure S9, Supporting Information), which is also consistent with the change in the ^1^H and ^17^O NMR spectra of the solvents in the electrolytes (Figure S10, Supporting Information). Furthermore, dynamic light scattering (DLS) analysis reveals a significant increase in the diameter of solvation clusters from 615 nm to 1280 nm and 2300 nm in the electrolyte incorporating 1.0 M, 2.5 M, and 5.0 M LiTFSI concentration (Figure S11, Supporting Information). Self‐diffusion analysis (Figure 3d) corroborates the formation of solvation clusters, where at 100% gradient pulse the solvent‐anion pair retains a much higher intensity of 60% from its intensity at 1% gradient pulse than DOL (4.2%) and DME (24.9%), indicating a larger molecular size of the pair compared to that of DOL and DME. Diffusion ordered spectroscopy analysis (Figure 3e) shows that the solvent‐anion pair (such as at 2.22 ppm in 2.5 M electrolyte) travels at a similar pace as DME with a diffusion coefficient of 6.0 × 10^–10^ m^2^ s^–1^, confirming that the solvent‐anion pair originates from the interactions of DME in the electrolytes. The findings are consistent as *δ* ^+^H of DME appears to be more electron‐deficient than that of DOL, which induces more favorable intermolecular pairs (Figure S12–S13, Supporting Information). Based on the results, the pairing of solvent and anion preferably appears with stronger electron‐deficient solvents and leads to the formation of conjugated solvation complexes, especially in the concentrated electrolytes.

The schematic microstructure of the solvation structure is presented to elucidate the above findings of the properties of the pairs (Figure 3f). The constructed model takes into account the convention of first solvation sheath of Li^+^ and the formation of ion pairs with increasing salt concentration, where the anion‐cation interactions so‐called contact ion pair and aggregate ion pair increase due to a higher population of anion in the first solvation sheath (Figure S14, Supporting Information).^[^ ^6b^ ^]^ The solvent and anion interactions are inversely proportional to the mole ratio of the solvents and the anion,^[^ ^17^ ^],^ i.e., (*x*+*y*):*z* in the aforementioned formula of solvation structure. The pairs are enhanced while the solvent/anion ratio decreases from 11.97:1, 4.79:1, 2.40:1, and 1.60:1 in the electrolyte concentration of 1.0, 2.5, 5.0, and 7.5 M, respectively, since the scarce number of solvents around Li^+^ enables the anion to enter the first solvation sheath and interact with *δ* ^+^H. The results indicate that the *δ* ^+^H‐anion pairs in the higher electrolyte concentration tend to bridge the originally discrete solvation sheaths and lead to the formation of a conjugated solvation network in the extended range, which could be responsible for the exponentially increased viscosity of the electrolytes (Figure S15, Supporting Information) and weakened/de‐shielded Li^+^ solvation (Figure S16a, Supporting Information). The latter particularly serves as the fundamental origin of easier de‐solvation and faster transfer of cation than that of anion,^[^ ^6^, ^11^, ^18^ ^]^ as well as the unusual interphasial chemistry, as evidenced by the reversible Li^+^ intercalation/de‐intercalation at the graphitic electrode when the LiTFSI concentration is above 5.0 M, or when the chemical shift of the ^1^H‐nuclei in solvent‐anion pair is below 4.11 ppm (Figure 3g), which is otherwise impossible for ether‐based electrolytes at diluted LiTFSI concentrations.^[^ ^6^, ^19^ ^]^ The finding is in good agreement with previous reports on the effect of Li^+^ coordination on the stability of graphite.^[^ ^6^, ^20^ ^]^ Li^+^ stripping‐plating in the electrolytes of high salt concentrations also benefits from such extended liquid structures (Figure 3h) In particular, the improved cycling stability and suppressed polarization of Li^+^ stripping‐plating are achieved upon increasing the salt concentration. The improved performances should correspond to the enhanced Li^+^ ions transfer at the electrolyte‐electrode interfaces,^[^ ^21^ ^]^ which could be due to the formation of a conjugated solvation network in the electrolytes as a result of enhanced solvent and anion interactions that are promoted by the high concentration of the anion. The observation not only completes the picture of electrolyte microstructure besides the known Li^+^ solvation and formation of ion pairs in the first solvation sheath,^[^ ^6^, ^18^ ^]^ but also paves the way to a better understanding of relationships between the electrolyte microstructure and battery performances.

**Figure 4:**
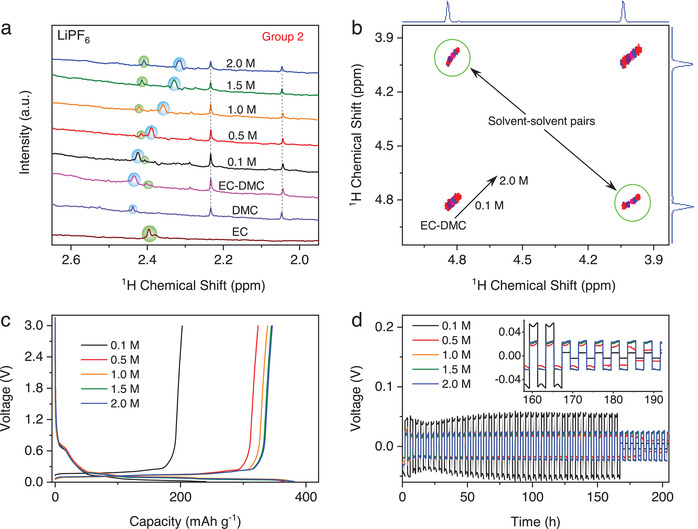
Solvent and anion pairs in the carbonate‐based electrolytes. a) ^1^H NMR and b) COSY spectra showing the solvent‐anion and solvent‐solvent pairs in the LiPF_6_ EC‐DMC electrolytes. c) Charge–discharge curve of Li vs. graphite cells and d) Li^+^ stripping‐platting curve of Li vs. Li symmetric cells. The pairs become more shielded with higher salt concentration, followed by a progressive improvement in the Li^+^ intercalation/deintercalation at graphite as well as Li^+^ stripping‐plating process.

Similar pair formations were also found in typical carbonate‐based electrolytes that have been extensively used in commercial Li‐ion batteries,^[^ ^22^ ^]^ such as LiPF_6_ in ethylene carbonate (EC) and dimethyl carbonate (DMC) 1:1 volume ratio or 1.27:1.0 molar ratio. We confirm again that the environment in the electrolyte changes upon Li^+^ solvation process (Figure S16b and S17, Supporting Information). The ^1^H NMR spectra of the electrolyte reveal a solvent‐anion pair from Group 2 in the region of 2.5–2.2 ppm that originates from DMC (inside blue circle in **Figure** **4**a). The pairing becomes more shielded as the concentration of LiPF_6_ increases from 0.1 M electrolyte (2.42 ppm) to 2.0 M (2.31 ppm). In particular, the solvent‐anion pair travels in the 1.0 M electrolyte at a diffusion coefficient of 5.78 × 10^–10^ m^2^ s^–1^ (Figure S18, Supporting Information). Moreover, the pair associated with EC becomes de‐shielded, as ^1^H‐nuclei turn into more electron‐deficient as a result of the strong Coulombic interaction between Li^+^ cation and the carbonyl‐O in the EC (inside green circle in Figure 4a), which apparently arises from the preferential cation‐solvent interaction between Li^+^ and EC.^[^ ^2^, ^8^ ^]^ The peaks at 2.04 and 2.23 ppm originated from DMC remain static despite the salt concentration increases, which suggests a selective interaction between the solvent and anion. Interestingly, despite the significant presence of EC in the electrolyte, the solvent‐anion pairing preferentially occurs with DMC rather than EC. This disparity agrees well with the previous finding that Li^+^ is preferentially solvated by EC, while DMC prefers anion solvation due to the more accessibility of the partial positive charge on the carbonyl.^[^ ^23^ ^]^ We have also observed the solvent‐solvent pair in the neat EC‐DMC mixture (Figure S19a,b, Supporting Information), which results from hydrogen bonding that underpins the dissolution of the solid EC in the liquid DMC (Figure S19c, Supporting Information). Such solvent‐solvent interaction becomes intensified as revealed by the more shielded ^1^H‐nuclei in the electrolytes at increasing LiPF_6_ concentration (Figure 4b). The formation of solvent–solvent interaction and solvent‐anion pairs in the electrolytes shows a strong correlation with the interphasial chemistries on both graphitic anode (Figure 4c) and lithium‐metal anode (Figure 4d). In particular, Li^+^ intercalation at graphite and Li^+^ stripping‐plating at Li metal are progressively improved when the pairs are more shielded. The analysis is consistent with the results in Figure 3g,h, where the pairs induce the formation of a conjugated solvation network in the electrolytes and subsequently improve the Li^+^ ions transfer. Thus, the solvent and anion pairs in the solvation sheaths appear as a potential probe to elucidate the ion transport behaviors within bulk electrolytes and across the electrolyte‐electrode interfaces.

## 3 Conclusion

This study has revealed the existence of hitherto unknown weak but long‐ranged solvent‐anion and solvent–solvent interactions in battery electrolytes. Such interactions arise from the induced hydrogen bonding (*δ* ^+^H) in the solvent molecules and either other solvent molecules or negatively‐charged anions. The pairs cast the formation of a conjugated solvation network in the electrolytes, which are strongly dependent on the electrolyte components (solvents, additives, salts, and concentration), which in turn dictate the ion transport within bulk electrolytes and across the electrolyte‐electrode interphases. The precise identification of these new interactions sets the foundation for the rational design of better electrolytes, e.g., by promoting solvent and anion pairs in the electrolytes. More importantly, the methodology established in this work significantly advances our understanding of how electrolyte components interact and arrange themselves, going beyond the commonly known cation solvation and cation‐anion pairings. Thus, our findings complete the picture of interactions in a complicated liquid system, which is of universal significance to the study of solvation behaviors and their relationships with battery performances.

## Conflict of Interest

The authors declare no conflict of interest.

## Supporting information

Supporting Information

## Data Availability

Research data are not shared.
