# Supplementary material for: Hitherto Unknown Solvent and Anion Pairs in Solvation Structures Reveal New Insights into High‐Performance Lithium‐Ion Batteries
Source: Adv Sci (Weinh). 2022 Aug 17;9(28):2202405. doi: 10.1002/advs.202202405 (PMC9534968; doi:10.1002/advs.202202405)
Supplement: Supplementary file 1 — Supporting Information [file ADVS-9-2202405-s001.pdf]

ADVANCED SCIENCE
Open Access

Supporting Information

for *Adv. Sci.*, DOI 10.1002/advs.202202405

Hitherto Unknown Solvent and Anion Pairs in Solvation Structures Reveal New Insights into High-Performance Lithium-Ion Batteries

Wandi Wahyudi, Xianrong Guo, Viko Ladelta, Leonidas Tsetseris, Mohamad I. Nugraha, Yuanbao Lin, Vincent Tung, Nikos Hadjichristidis, Qian Li, Kang Xu, Jun Ming* and Thomas D. Anthopoulos**

Supporting Information

Hitherto Unknown Solvent and Anion Pairs in Solvation Structures Reveal New Insights into High-Performance Lithium-Ion Batteries

Wandi Wahyudi,[†] Xianrong Guo,[†] Viko Ladelta,[†] Leonidas Tsetseris, Mohamad I. Nugraha, Yuanbao Lin, Vincent Tung, Nikos Hadjichristidis, Qian Li, Kang Xu, Jun Ming,* Thomas D. Anthopoulos**

Dr. W. Wahyudi, Dr. M. I. Nugraha, Dr. Y. Lin, Prof. V. Tung and Prof. T. D. Anthopoulos
KAUST Solar Center, King Abdullah University of Science and Technology (KAUST), Thuwal 23955-6900, Saudi Arabia
E-mail: thomas.anthopoulos@kaust.edu.sa

Dr. Q. Li and Prof. J. Ming
State Key Laboratory of Rare Earth Resource Utilization, Changchun Institute of Applied Chemistry, Chinese Academy of Sciences, Changchun 130022, People's Republic of China
E-mail: jun.ming@ciac.ac.cn

Dr. V. Ladelta and Prof. N. Hadjichristidis
KAUST Catalysis Center, King Abdullah University of Science and Technology (KAUST), Thuwal 23955-6900, Saudi Arabia

Dr. X. Guo
Core Labs, King Abdullah University of Science and Technology (KAUST), Thuwal 23955-6900, Saudi Arabia

Dr. M. I. Nugraha
Research Center for Advanced Materials, National Research and Innovation Agency (BRIN), South Tangerang, Banten 15314, Indonesia

Prof. L. Tsetseris
Department of Physics, National Technical University of Athens, Athens GR-15780, Greece

Prof. K. Xu
Battery Science Branch, US Army Research Laboratory, Adelphi, Maryland 20783, United States
E-mail: conrad.k.xu.civ@mail.mil

[†]These authors contributed equally to this work.

Keywords: electrolytes, solvation structure, nuclear magnetic resonance (NMR) spectroscopy, lithium-ion batteries, solvent and ion pairs

Experiments

Materials and preparation. Lithium bis(trifluoromethanesulfonyl)imide ($LiNC_2F_6S_2O_4$, LiTFSI), lithium trifluoromethanesulfonate (($LiCF_3SO_3$, LiTf), lithium hexafluorophosphate ($LiPF_6$), lithium nitrate ($LiNO_3$), 1,3-dioxolane (DOL), 1,2-dimethoxyethane (DME), ethylene carbonate (EC), and dimethyl carbonate (DMC) were purchased from Sigma Aldrich. Deuterium oxide (D_2O), and dimethyl sulfoxide-d6 (DMSO-d6) were purchased from (Cambridge Isotope Laboratory, USA). The electrolyte solutions were prepared in an argon-filled glove box at room temperature (23°C), where the stoichiometric ratio of lithium salt was added into the solvent(s) in the vial and stirred with a magnetic stirrer.

Liquid NMR experiments. All NMR tubes were dried in a vacuum at 60°C before use. In the glovebox, the electrolyte (400 µL) was transferred into the NMR tube (5 mm thin wall 7" 1000 MHz, Wildmad). The 100 µL NMR solvent (deuterium oxide, D_2O,) was filled into a coaxial NMR insert (Wildmad) and then inserted into the NMR tube. Both the NMR tube and the NMR insert were tightly sealed with their caps and Parafilm tape. The NMR insert was cleaned with the neat solvent mixture and then dried and re-used for analysis of the other electrolytes. The NMR experiment was performed at 25°C on a Bruker Avance III 600 MHz liquid NMR spectrometer equipped with a Z-axis gradient filed (50 G/cm in maximum) BBFO probe. The ^{1}H NMR spectra were acquired using typical standard parameters of one pulse sequence with a recycle delay of 2 s, a receiver gain of 2.56, and 80 number of scans. The ^{1}H-^{1}H correlation spectroscopy (COSY) spectra were collected with 4096 points in the direct detecting dimension (t2) and 512 points in the indirect detecting dimension (t1) over a spectral width of 12.0 ppm in both dimensions, with eight scans per t1 point. NMR measurement of ethylene carbonate (EC) solvent was performed under mild heating at 35°C. All the ^{1}H NMR and COSY spectra were referenced to the D_2O residual proton signal at 4.79 ppm. The ^{17}O NMR spectra were acquired with a spectral width of 400.0 ppm, a pulse duration of 12 µs, an acquisition time of 0.25 s, a recycle delay of 200 ms, a receiver gain of 203, and 12288 scans. All of the ^{17}O spectra were referenced to the ^{17}O signal of D_2O at 0.0 ppm. The ^{1}H (1D) self-diffusion spectra were recorded by a double stimulated echo sequence with 3 spoil gradients, along with a diffusion time of 0.3, 0.7, or 0.8 s, a gradient pulse of 1000 us length and SMSQ10 shape a receiver gain of 9.0, a spectra width of 12.0 ppm over 65536 points collected, and 512 scans, for both 1% and 100% gradient pulses.

Solvent suppression ^{1}H NMR spectra was collected by Bruker's p3919gp pulse program with a recycle delay of 2 s, a receiver gain of 203, and 80 number of scans. A 3-9-19 pulse

sequence with gradients was applied to suppress both of the solvent peaks by adjusting the delay for the binomial solvent suppression time to 2 ms. The ^{1}H diffusion-ordered spectroscopy (DOSY) spectra were recorded using the Bruker's stebpgp1s19 pulse program with a diffusion time (D) of 0.3 s and gradient length (δ) of 1000 µs, with 65536 points in the direct detecting dimension (t2), 16 gradient strength values increasing linearly from 2% to 98%, 512 scans per gradient strength value, and a receiver gain of 203. The 90° pulse width was measured before running the DOSY experiment, and the spectra were recorded with a recycle delay of 2 s between scans. T1 was evaluated independently to guarantee sufficient relaxation of the magnetization during the recycle delay. A 3-9-19 pulse sequence with gradients was also used as the ^{1}H NMR solvent suppression experiment. The data were processed using the DOSY software within the Topspin 3.5 software package, with a non-line least-squares fitting algorithm applied.

Other characterizations. Thermogravimetry and differential scanning calorimetry (TG-DSC) analysis were carried out using a Simultaneous Thermal Analyzer (STA 449 F1, Netzsch) in an N_2 atmosphere with a heating rate of 20°C min^{-1} and a temperature range of 30°C to 600°C. Fourier transform infrared (FTIR) measurement was carried out using a Horiba spectrometer. Dynamic light scattering (DLS) analysis of the samples was performed using a Zetasizer Nano (Malvern Instruments) for around 180 number of scans using glass cuvettes. The measurement was carried out with 1.0 M LiTFSI in DOL-DME electrolyte as the reference, with a refractive index of 1.395 (obtained from Reichert refractometer) and absorption of 0.002 (obtained from Varian UV Vis spectrometer). The dispersant was a DOL-DME mixture 1:1 volume ratio, with a viscosity of 0.6307 cp (25°C) measured by Kyoto Electronics, a refractive index of 1.390, and a dielectric constant of 7.16. Raman spectra of the electrolytes were collected by employing Witec alpha 300 Raman spectrometer with an exciting laser of 532 nm, using quartz cuvettes that were tightly sealed in an argon-filled glovebox. Deconvolution of the $TFSI^-$ S-N band was performed by using XPS Peaks software with Shirley type background and $TFSI^-$ coordination bands of solvent separated ion pair (SSIP) at 741 cm^{-1}, contact ion pairs (CIP) at 744 cm^{-1}, and aggregate ion pairs (AIP) at 747 cm^{-1}. The viscosity of the electrolytes was measured with a viscometer (Kyoto Electronics) at 25°C. The ionic conductivity of electrolytes was measured with an impedance analyzer (VMP3 Biologic) at room temperature.

Density functional theory (DFT) calculations. DFT calculations were performed with the code NWChem, the B3LYP exchange-correlation functional, and the DZVP orbital basis.[1-4] Van der Waals interactions were included with the so-called DFT-D3 method.[5]

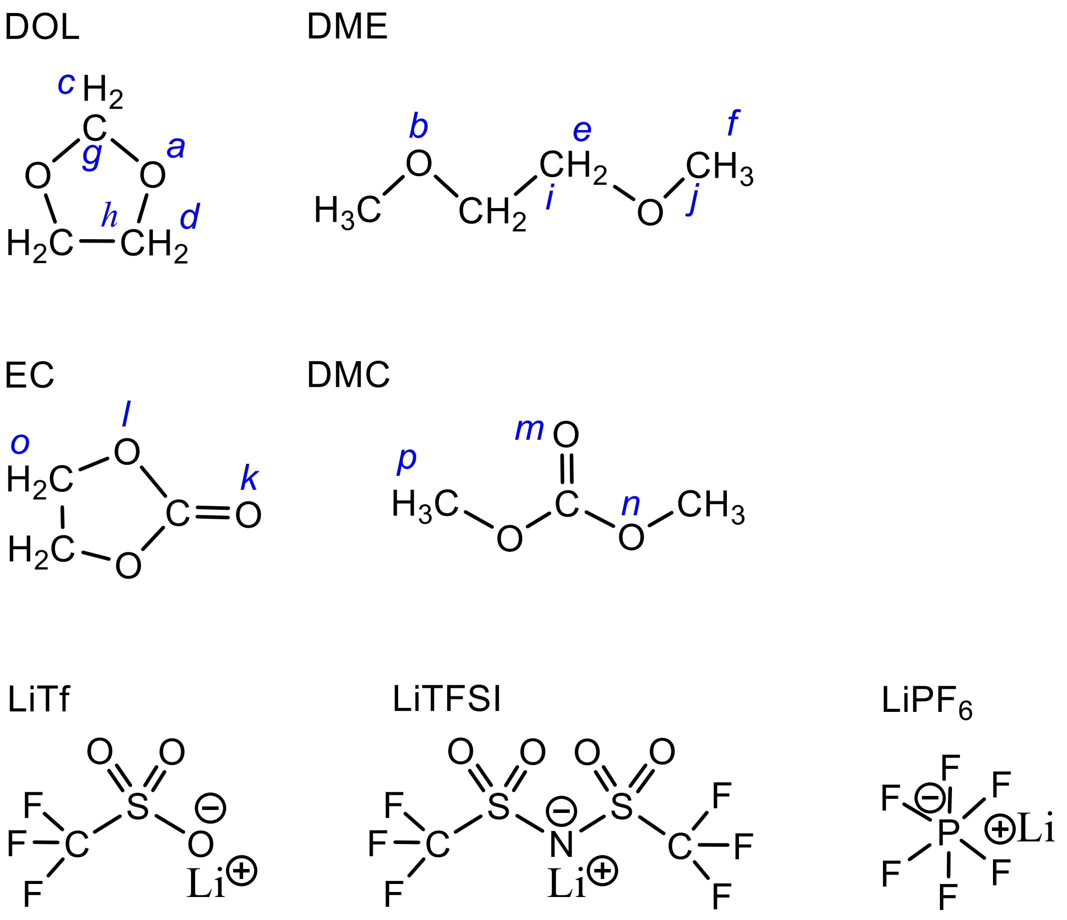

Figure S1. Chemical structure of solvents and Li salts. The labels *a-p* (blue) near the atoms are used to indicate the chemical shift of the nuclei in the NMR spectra.

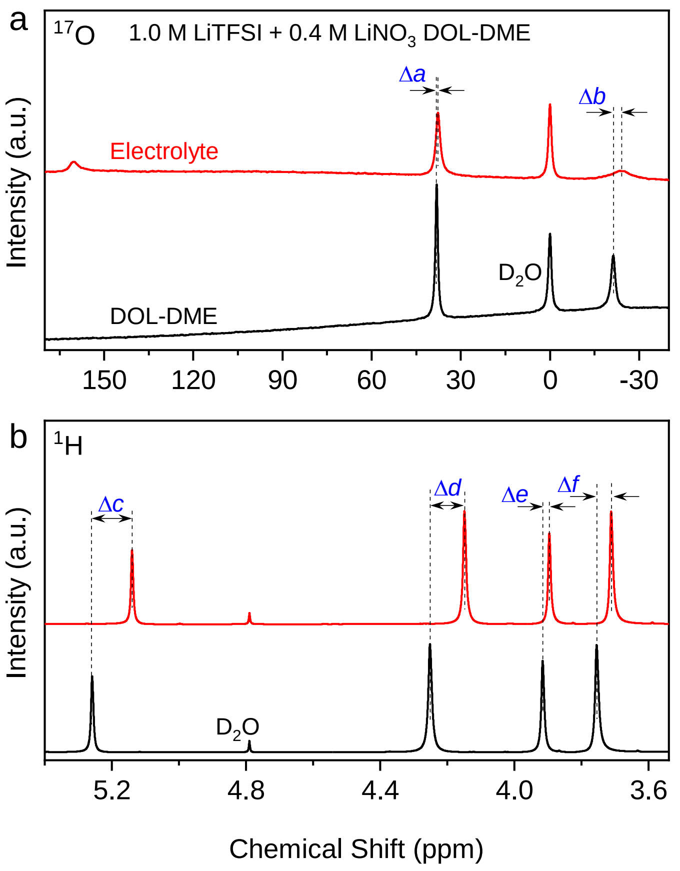

Figure S2. Changes of the solvent environment in the ether-based electrolytes. (a) ^{17}O and (b) ^{1}H NMR spectra of the neat DOL-DME and the electrolyte incorporating 1.0 M LiTFSI and 0.4 M $LiNO_3$ salts. The oxygen and hydrogen nuclei of DOL and DME in the electrolyte are shielded compared to the neat DOL-DME mixture, indicating the varied solvent environment upon the Li^+ solvation process. The peak assignments and chemical shift displacements are listed and summarized in **Figure S1** and **Table S1**, respectively.

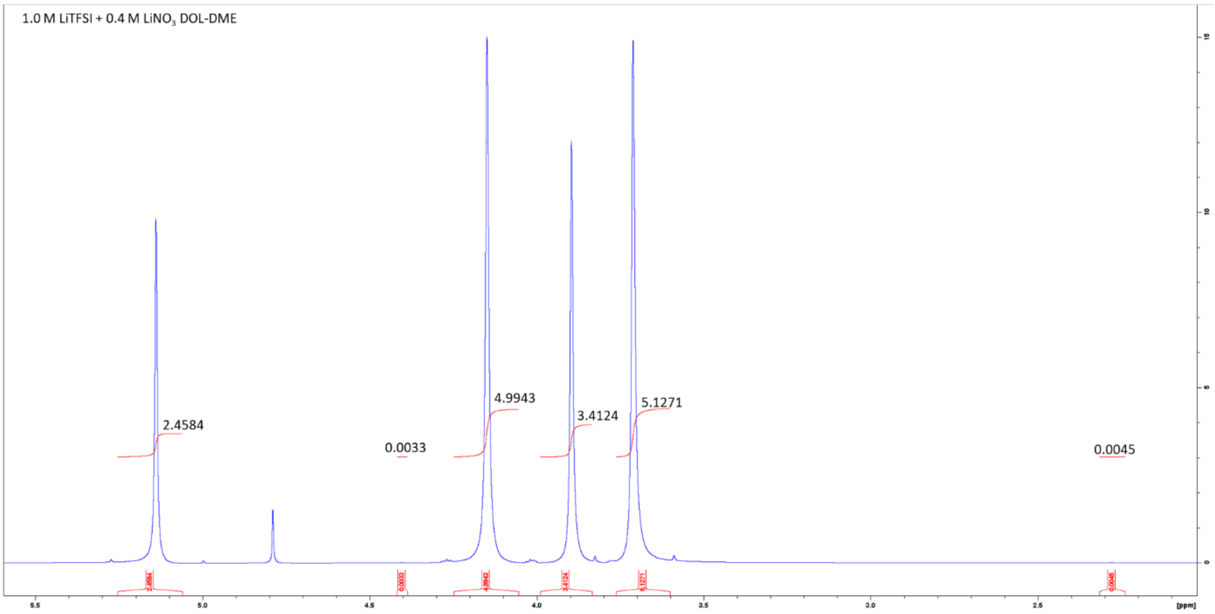

Figure S3. Ratio of integral of the peaks in the ^{1}H NMR spectra from 1.0 M LiTFSI + 0.4 M $LiNO_3$ DOL-DME electrolyte. By inserting the total number of the hydrogen atom in each DOL and DME molecule of 16 into the integral ratio, the new peaks at 4.41 ppm and 2.28 ppm have the integral ratio for a hydrogen atom of 0.0033 and 0.0045, respectively.

Table S1. Changes of the solvent environment in the electrolytes. Displacement of ^{1}H and ^{17}O spectra of solvents in the electrolytes with different compositions at 25°C, calculated with respect to the spectra of corresponding solvent mixture.

**Electrolyte**	**Chemical Shift (ppm)**					
	**^{17}O DOL**	**^{17}O DME**	**^{1}H DOL**		**^{1}H DME**	
	Δ*a*	Δ*b*	Δ*c*	Δ*d*	Δ*e*	Δ*f*
LiTFSI + $LiNO_3$ DOL-DME	-0.429	-2.71	-0.1186	-0.1026	-0.0197	-0.0429
1.0 M LiTFSI DOL	-0.746		-0.0544	-0.0416		
1.0 M LiTFSI DME		-1.46			-0.117	-0.1306
1.0 M LiTf DOL-DME	-0.336	-1.72	-0.0772	-0.0699	-0.0276	-0.0372
1.0 M $LiPF_6$ DOL-DME	0.046	-2.49	-0.124	-0.1055	-0.0341	-0.0515
1.0 M LiTFSI DOL-DME	-0.134	-2.41	-0.1194	-0.1058	-0.0319	-0.0485
2.5 M LiTFSI DOL-DME	-0.48	-4.23	-0.2032	-0.1815	-0.0673	-0.0938
5.0 M LiTFSI DOL-DME	-0.26		-0.2305	-0.1988	-0.1308	-0.1573
6.0 M LiTFSI DOL-DME			-0.2315	-0.1951	-0.1553	-0.1805
7.5 M LiTFSI DOL-DME			-0.2428	-0.2016	-0.1926	-0.2171
**Electrolyte**	**^{17}O EC**		**^{1}7O DMC**		**^{1}H EC**	**^{1}H DMC**
	Δ*k*	Δ*l*	Δ*m*	Δ*n*	Δ*o*	Δ*p*
0.1 M $LiPF_6$ EC-DMC	-0.88	0.21	-0.49	0.14	-0.0004	-0.0024
0.5 M $LiPF_6$ EC-DMC	-4.66	1.13	-2.76	0.48	-0.0102	-0.0215
1.0 M $LiPF_6$ EC-DMC	-10.92	2.78	-7.83	1.43	-0.0314	-0.0518
1.5 M $LiPF_6$ EC-DMC	-12.86	3.3	-9.46	2.04	-0.0372	-0.0596
2.0 M $LiPF_6$ EC-DMC	-17.2	3.9	-14.2	3.4	-0.0474	-0.0711

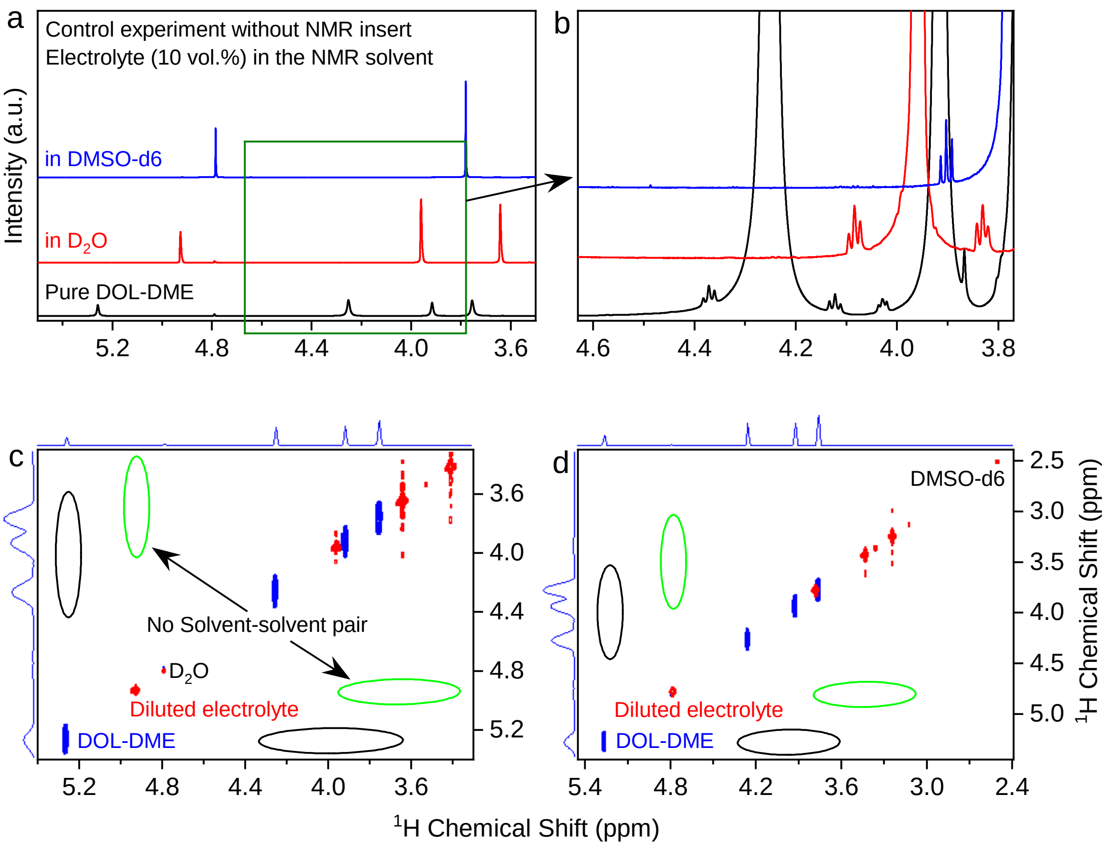

Figure S4. Control experiment. The electrolyte was diluted (10 vol. %) in the D_2O and DMSO-d6 NMR solvents, which follows the routine technique of a standard liquid NMR experiment. (a, b) ^1H and (c, d) 2D COSY spectra of the electrolytes. In detail, 50 μL of electrolyte (e.g., 1.0 M LiTFSI, 0.4 M $LiNO_3$ in DOL-DME) was mixed with 450 μL of NMR solvent (D_2O or DMSO-d6) in a vial, which was then transferred into the NMR tube and sealed for NMR analysis. The references for the ^1H spectra were 4.79 ppm and 2.49 ppm that corresponding to ^1H in D_2O and DMSO-d6, respectively. The displacement of the ^1H NMR spectra can be seen between the diluted electrolytes in the different NMR solvents (**Figure S4**a), indicating a significant change in the environment of DOL and DME affected by the NMR solvents. Furthermore, the solvent-anion (**Figure S4**b) and solvent-solvent interactions are invisible in both the diluted electrolytes in D_2O (**Figure S4**c) and DMSO-d6 (**Figure S4**d) NMR solvents. The observation demonstrates that the NMR solvents interact with the electrolytes and then change the solvation structure, showing the importance of preserving the nature of the electrolytes during analysis with NMR spectroscopy, *i.e.*, by using an internal insert.

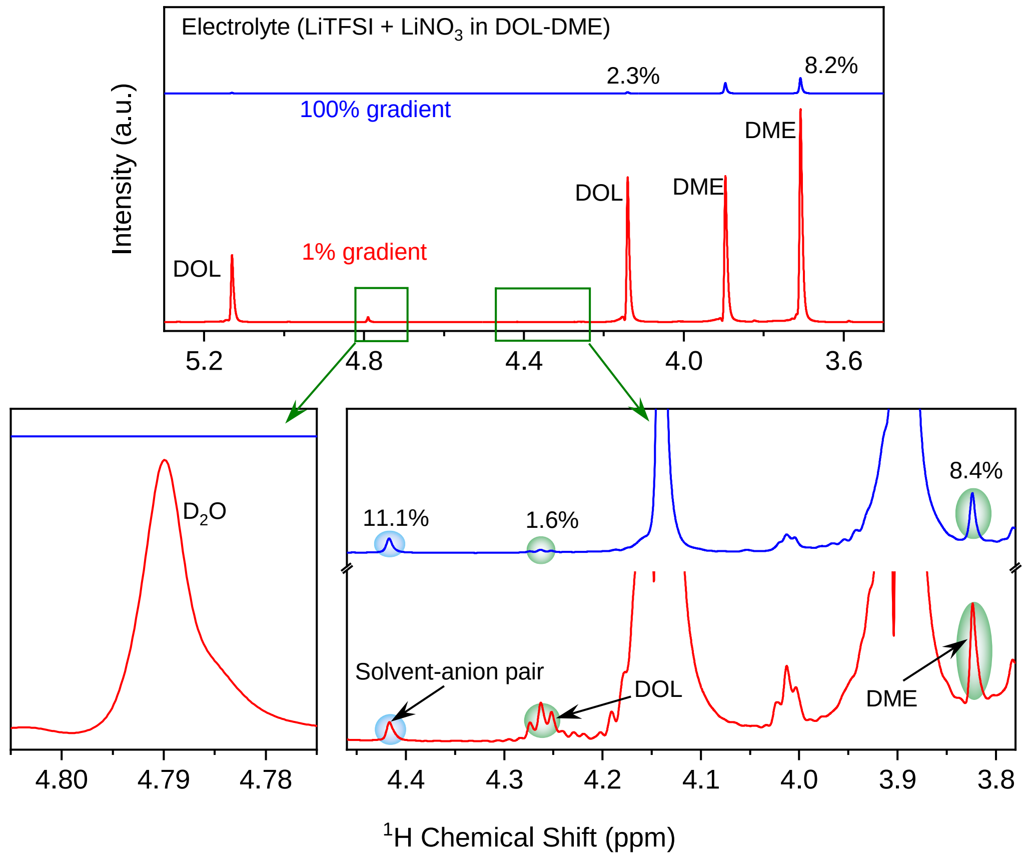

Figure S5. Self-diffusion analysis of the electrolyte. 1D spectra of ^1H self-diffusion of the electrolyte (1.0 M LiTFSI, 0.4 M $LiNO_3$ in DOL-DME) measured with a diffusion time (D20) = 0.3 second, outset: D_2O and solvent-anion pair regions. The solvent-anion pair at a 100% gradient pulse retains a much higher intensity (with the highest retention of 11.1% intensity from the spectra at a 1% gradient pulse) than DOL (retention of 2.3% intensity) and DME (retention of 8.2% intensity), indicating a slower diffusion rate and larger molecular size for the solvent-anion pair than that of DOL and DME.

Table S2. Composition of the electrolytes. The electrolytes were prepared with the following composition, in the Ar-filled glovebox.

Electrolyte	Quantity per 1 ml solution (gram)				Quantity (mole)				Mole Ratio
	**DOL**	**DME**	**Salt**	**$LiNO_3$**	**DOL**	**DME**	**Salt**	**$LiNO_3$**	**Solvent : Salt**
1.0 M LiTFSI, 0.4 M $LiNO_3$	0.530	0.4335	0.287	0.028	0.0072	0.0048	0.0010	0.0001	10.92 :1
1.0 M LiTFSI	0.530	0.4335	0.287		0.0072	0.0048	0.0010		11.97 : 1
2.0 M LiTFSI	0.530	0.4335	0.574		0.0072	0.0048	0.0020		5.98 : 1
2.5 M LiTFSI	0.530	0.4335	0.718		0.0072	0.0048	0.0025		4.79 : 1
3.0 M LiTFSI	0.530	0.4335	0.861		0.0072	0.0048	0.0030		3.99 : 1
4.0 M LiTFSI	0.530	0.4335	1.148		0.0072	0.0048	0.0040		2.99 : 1
5.0 M LiTFSI	0.530	0.4335	1.435		0.0072	0.0048	0.0050		2.40 : 1
6.0 M LiTFSI	0.530	0.4335	1.723		0.0072	0.0048	0.0060		1.99 : 1
7.5 M LiTFSI	0.530	0.4335	2.153		0.0072	0.0048	0.0075		1.60 : 1
1.0 M LiTf	0.530	0.4335	0.156		0.0072	0.0048	0.0010		11.97 : 1
1.0 M $LiPF_6$	0.530	0.4335	0.152		0.0072	0.0048	0.0010		11.97 : 1

Electrolyte	Quantity per 1 ml solution (gram)			Quantity (mole)			Mole Ratio
	**EC**	**DMC**	**Salt**	**EC**	**DMC**	**Salt**	**Solvent : Salt**
0.1 M $LiPF_6$	0.660	0.5345	0.015	0.0075	0.0059	0.0001	134.29 : 1
0.5 M $LiPF_6$	0.660	0.5345	0.076	0.0075	0.0059	0.0005	26.86 : 1
1.0 M $LiPF_6$	0.660	0.5345	0.152	0.0075	0.0059	0.0010	13.43 : 1
1.5 M $LiPF_6$	0.660	0.5345	0.228	0.0075	0.0059	0.0015	8.95 : 1
2.0 M $LiPF_6$	0.660	0.5345	0.304	0.0075	0.0059	0.0020	6.71 : 1

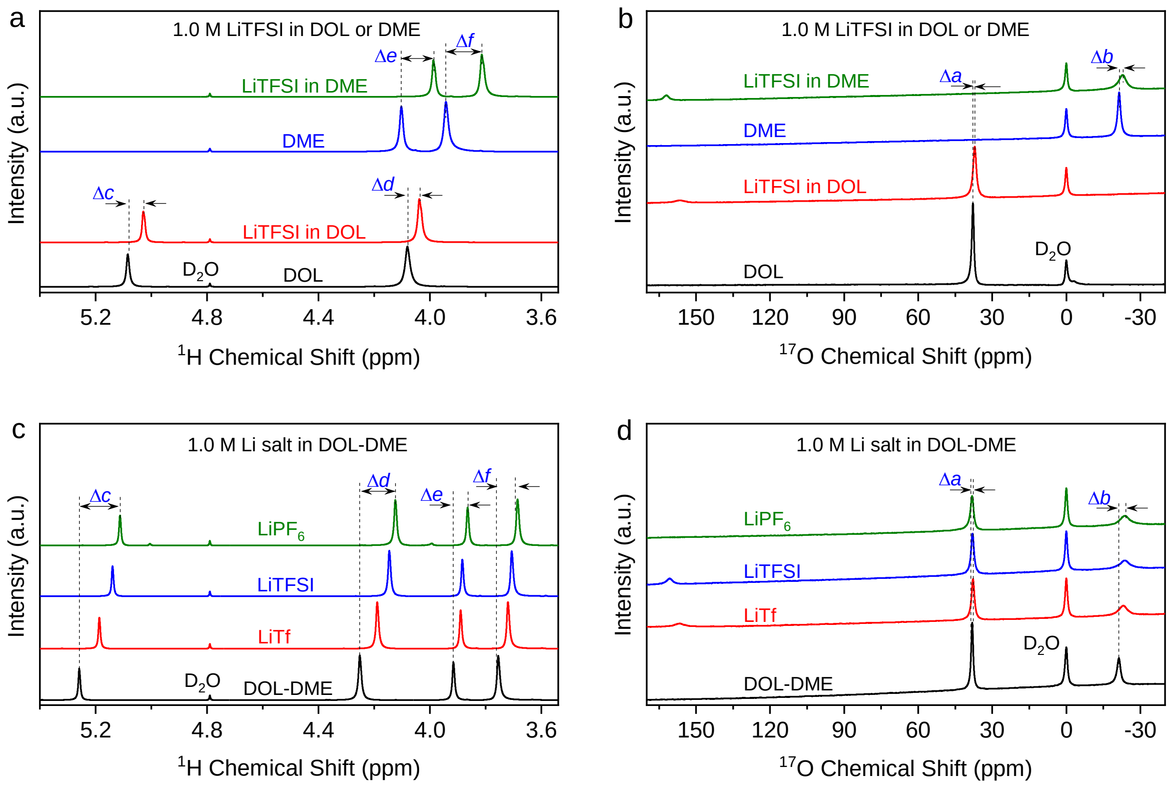

Figure S6. Solvent and anion dependencies of solvation sheaths. (a) ^{1}H and (b) ^{17}O NMR spectra of the electrolytes incorporating different solvents, *i.e.*, DOL and DME. (c) ^{1}H and (d) ^{17}O NMR spectra of the electrolytes incorporating different lithium salts, *i.e.*, LiTf, LiTFSI, and $LiPF_6$. The results show that the type of solvents and anions affect the solvation sheaths as indicated by the displacement of ^{1}H and ^{17}O chemical shifts in the electrolytes compared to the neat solvents.

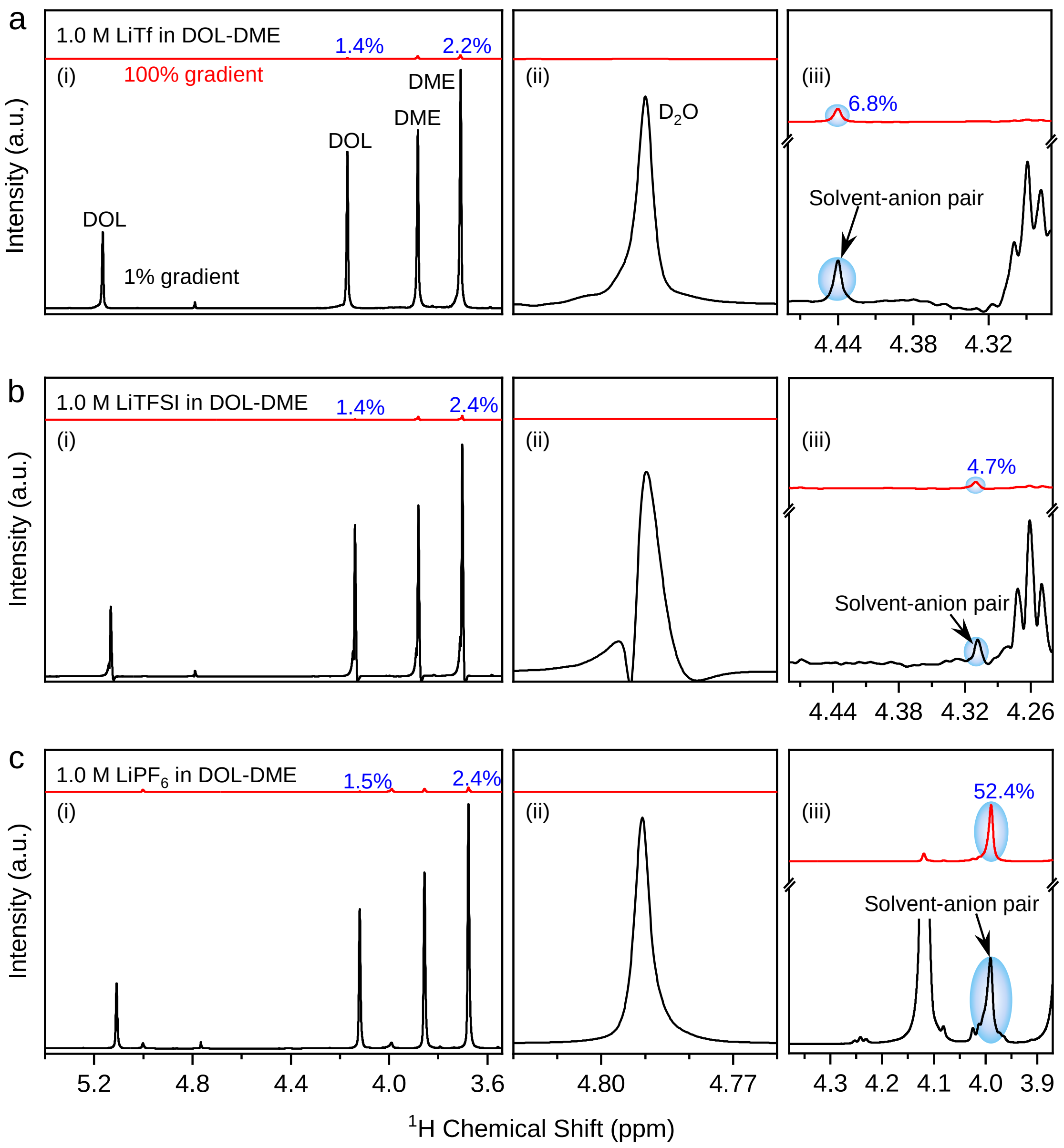

Figure S7. Self-diffusion analysis and Li salt-dependence of solvent-anion pairs. ^{1}H self-diffusion spectra of the electrolytes incorporating 1.0 M of (a) LiTf, (b) LiTFSI, and (c) $LiPF_6$ in DOL-DME. ^{1}H self-diffusion of (i) wide spectra, (ii) outset of D_2O and (iii) outset of solvent-anion pair and solvents regions. Diffusion time (D20) = 0.3 second. At 100% gradient pulse, the solvent-anion pair retains much higher intensity (with the highest retention of intensity from the spectra at a 1% gradient pulse) than the intensity of DOL and DME. Firstly, the results indicate that the solvent-anion pair diffuses with a slower diffusion rate, which suggests a larger molecular size of the pair than that of DOL and DME. Secondly, the solvent-anion pair in the $LiPF_6$ DOL-DME electrolyte diffuses much slower than that in the LiTf and LiTFSI systems, indicating the formation of much larger solvation complexes in the $LiPF_6$ DOL-DME electrolyte.

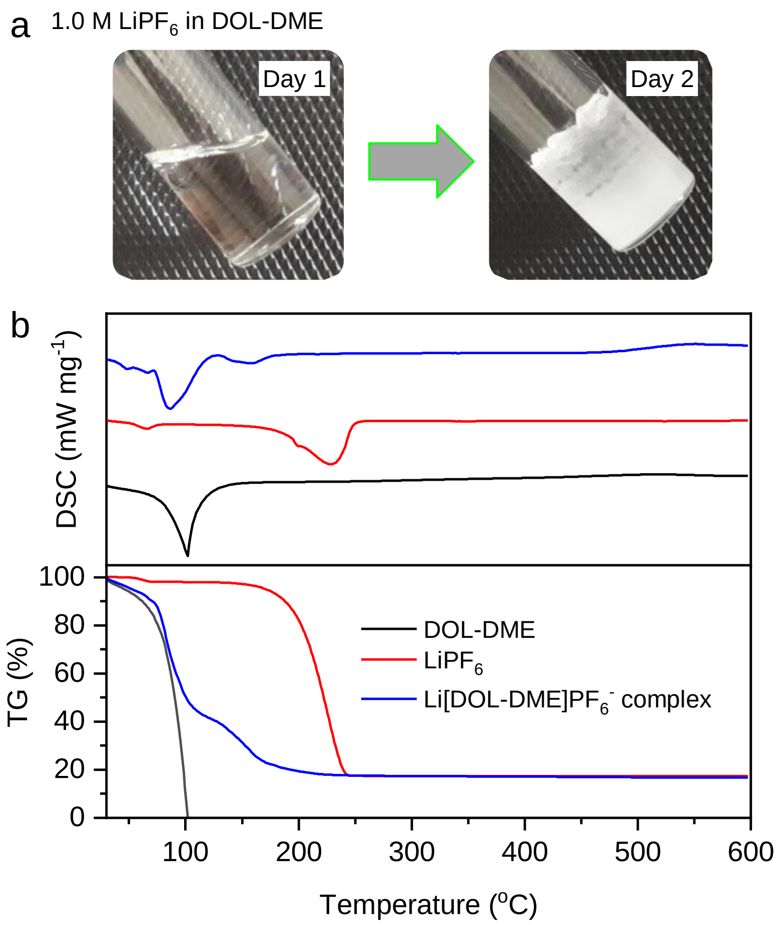

Figure S8. Properties of solvation complexes in the 1.0 M $LiPF_6$ DOL-DME electrolyte. (a) Photographs of the electrolyte incorporating 1.0 M $LiPF_6$ in DOL-DME show the phase transition from liquid to solid solvation complexes after one day of storage in the glovebox. (b) Thermal analysis (TG-DSC) of the electrolyte, $LiPF_6$ salt, and neat DOL-DME solvent shows different characteristics of the solvation complexes from the neat salt and solvents. This observation indicates the presence of strong solvent-anion interactions with simultaneous progress over time, providing further evidence of large solvation complexes induced by $LiPF_6$.

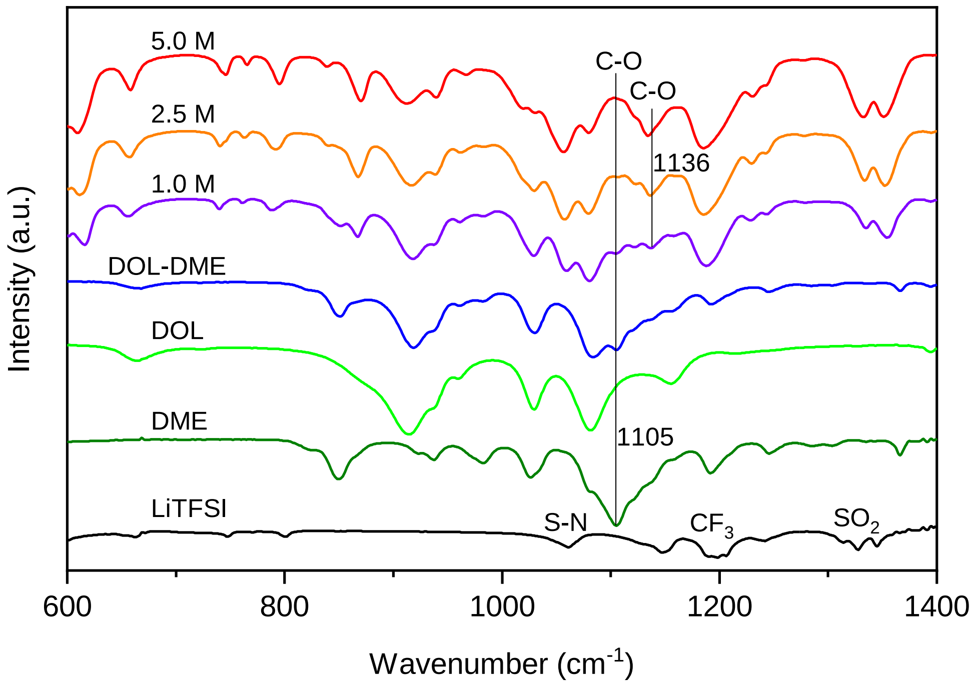

Figure S9. Microstructure of the solvation sheaths. Fourier transform infrared (FTIR) spectra of LiTFSI, DOL, DME, and their mixture in the electrolytes, showing that the C-O vibration band of DME at 1105 cm^{-1} disappears while a new C-O vibration band at 1136 cm^{-1} appears as the electrolyte concentration increases. The disappearance and new appearance of C-O vibrations in the electrolytes may underpin the formation of solvation clusters. The results suggest evidence of favorable intermolecular interactions involving DME.

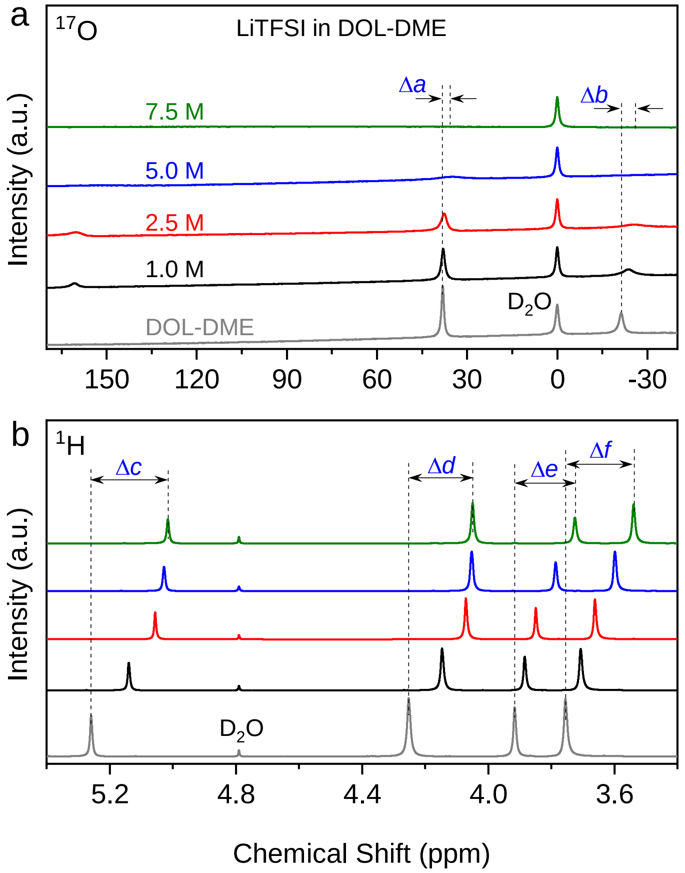

Figure S10. Concentration dependence of solvation sheaths. (a) ^{17}O and (b) ^{1}H NMR spectra of the electrolytes incorporating different LiTFSI concentration in DOL-DME. The ^{17}O NMR spectra of DOL and DME are invisible in the high concentration electrolytes, where the ^{17}O NMR spectra of DME disappear earlier, at 2.5 M LiTFSI concentration. The ^{1}H chemical shifts of DOL (*c* and *d*) are shielded and then saturated in the electrolyte higher than 5.0 M LiTFSI concentration. In contrast, the ^{1}H chemical shifts of DME (*e* and *f*) still show a simultaneous change, which indicates that DME is more sensitive to interactions with anions.

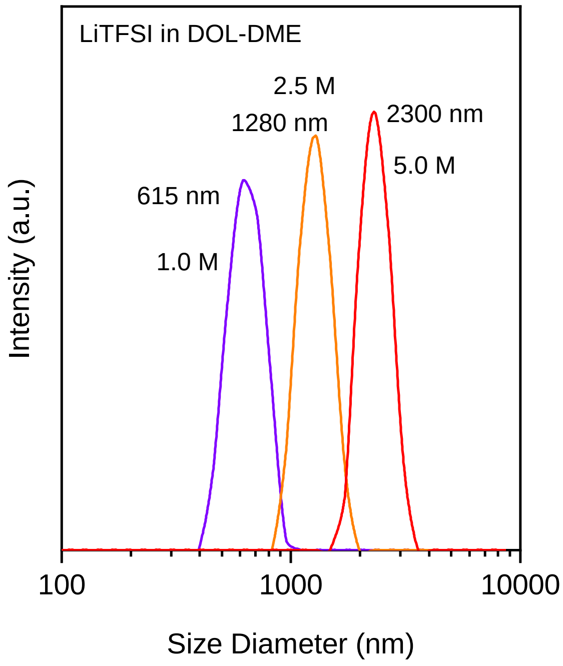

Figure S11. Changes in the size of solvation clusters. Dynamic light scattering (DLS) spectra of electrolytes showing a significant increase in the size of solvation clusters with higher LiTFSI concentration in the DOL-DME, *i.e.*, solvation cluster diameter of 615 nm (1.0 M) to 1280 nm (2.5 M) and 2300 nm (5.0 M). The result indicates that the cluster may appear with a distinguished boundary and exist for a long period without disturbance, which is also consistent with the slow decay of the solvent-anion peak (**Figure S7**) and observed gel-like complex (**Figure S8**).

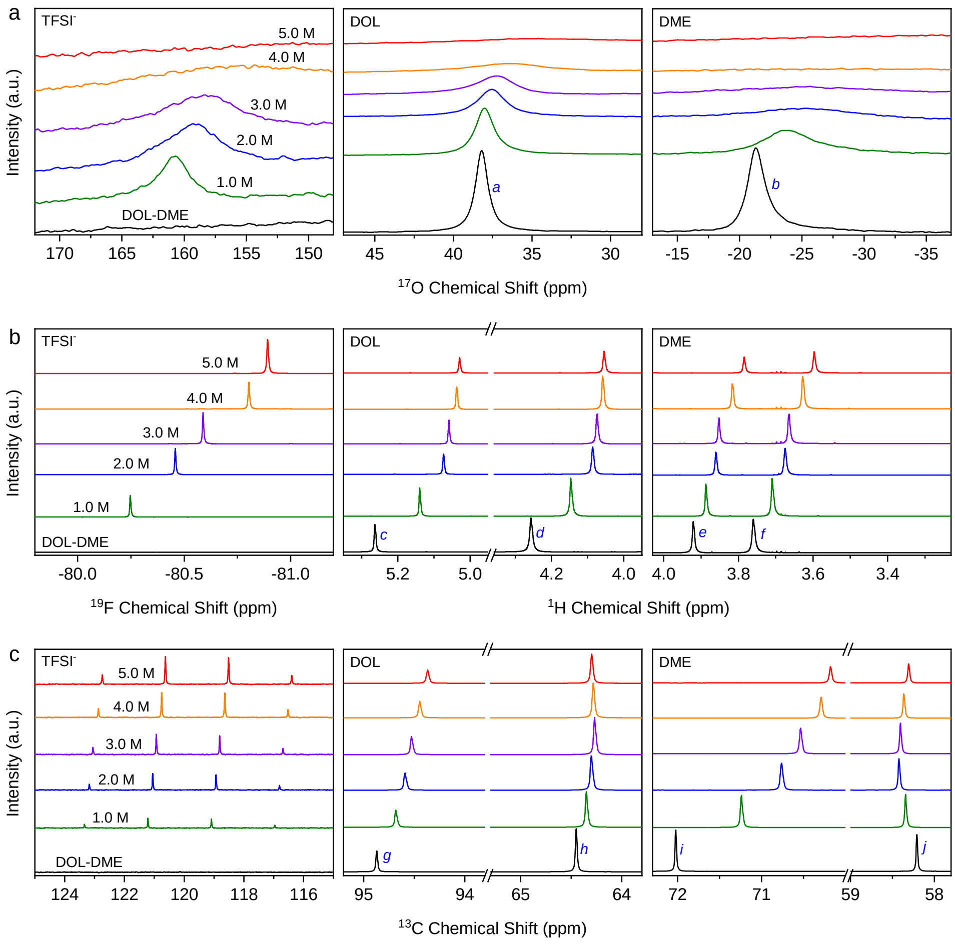

Figure S12. Plausible intermolecular pairs of solvents and anions in the electrolytes. (a) ^{17}O, (b) ^{19}F and ^{1}H, and (c) ^{13}C NMR spectra of electrolytes incorporating LiTFSI salt and DOL-DME solvents. The references were 0.1 M $LiPF_6$ in 1 vol.% H_2O + 10 vol.% C_2H_5OH + 98 vol.% D_2O using an internal NMR insert. The spectra were referred to ^{1}H of H_2O (4.84 ppm), ^{7}Li of $LiPF_6$ (0.107 ppm), ^{13}C of C_2H_5OH (57.446 ppm), ^{17}O of D_2O (0.0 ppm), and ^{19}F of $LiPF_6$ (-71.87 ppm).

Figure S12a shows that the ^{17}O spectra decay (broadened and then disappeared) in the high concentration electrolytes. The decay rate of NMR spectra should decrease from the largest to smallest molecules, *e.g.*, in the order of TFSI-, DME, and then DOL, due to the shorter transverse relaxation time of the NMR signals. However, the ^{17}O NMR spectra of DME broadens and decays earlier than $TFSI^-$ followed by DOL, the discrepancy should arise due to the formation of intermolecular pairs with preference to DME. The up-field shift of ^{19}F and ^{13}C NMR spectra of $TFSI^-$ shows a further evidence that the anion is shielded by electron clouds from the neighboring molecules such as DME and DOL solvents (**Figure S12**b, c). Furthermore, we find that the shift of ^{1}H NMR spectra of DOL is limited in the high electrolyte concentration, while the shift of ^{1}H NMR spectra of DME is more simultaneous (**Figure S12**b). The observation suggests an evidence of favorable hydrogen bonding between DME and $TFSI^-$, which also corroborates the strong electron-deficient hydrogen (δ^+H) in DME.

a b

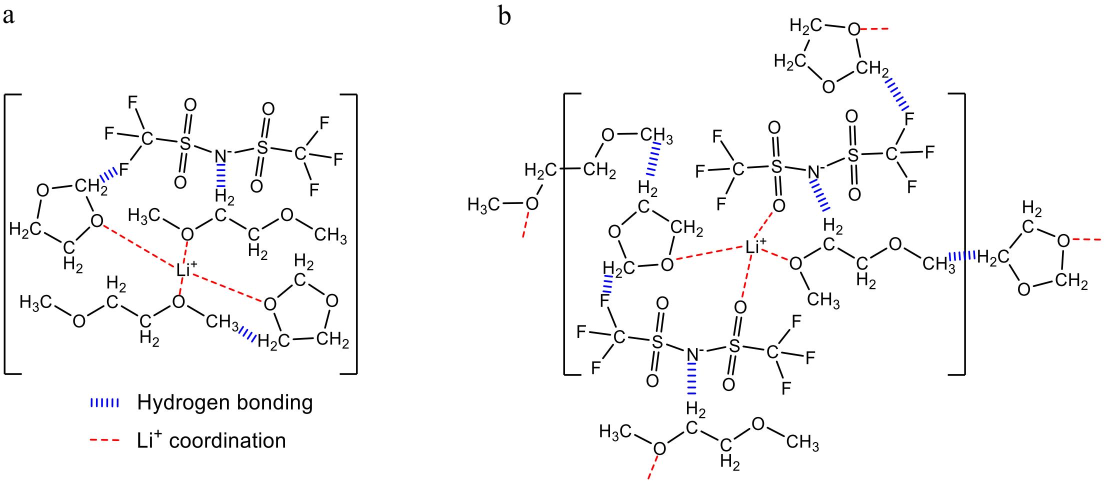

Solvation complex in the dilute electrolyte. Conjugated solvation complexes in the high concentration electrolyte.

Figure S13. Schematic plausible of intermolecular pairs in the electrolytes. Schematic of solvent-solvent and solvent-anion pairs in (a) a solvation complex in the low concentration electrolyte, and (b) the overlapping of adjacent solvation complexes in the high concentration electrolyte forms conjugated solvation complexes. The pairs in the low concentration electrolytes are localized within the solvation complex due to the excess of free solvent molecules. In the high concentration electrolytes, the formation of further pairs takes place between the neighboring solvation complexes, which are referred to as conjugated solvation complexes.

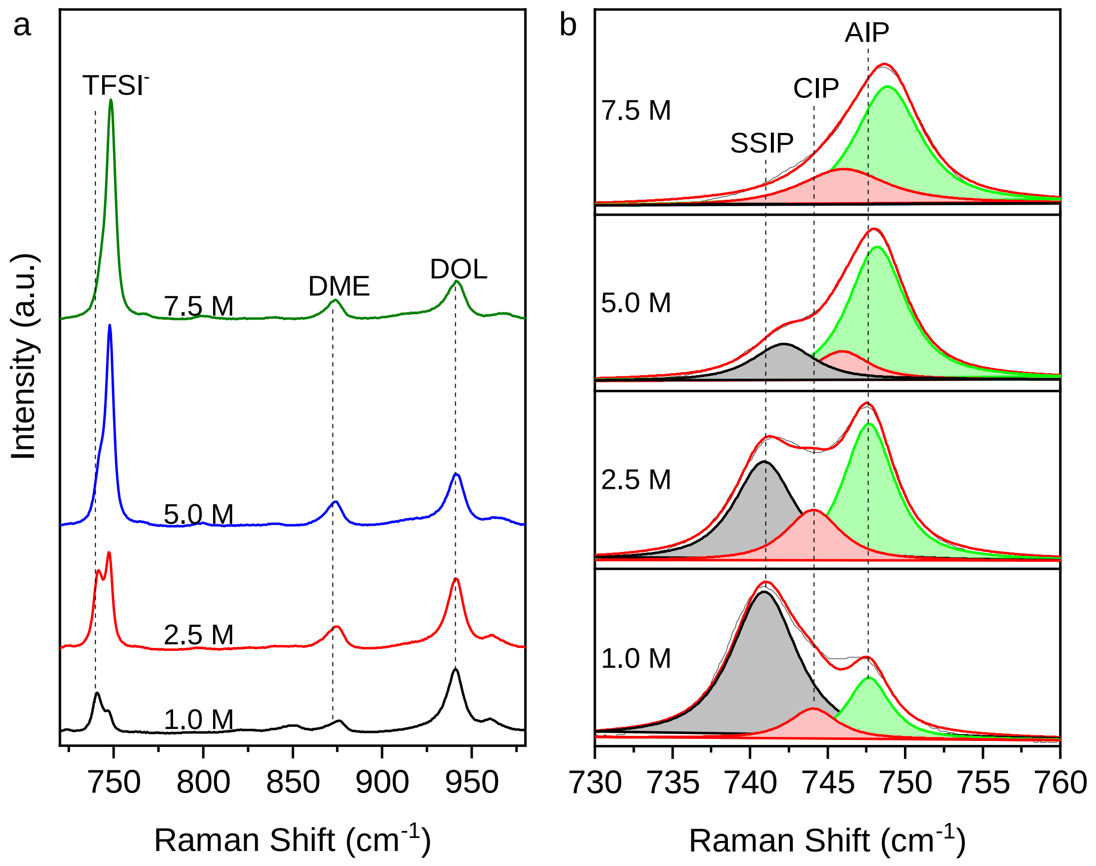

Figure S14. (a) Raman spectra of the DOL-DME-based electrolytes with increasing salt concentration, showing S-N stretching in $TFSI^-$ and C-O stretching in DME and DOL. (b) Deconvolution of S-N stretching band in $TFSI^-$ of the electrolytes, which represents solvent separated ion pair (SSIP) at 741 cm^{-1}, contact ion pair (CIP) at 744 cm^{-1}, and aggregate ion pair (AIP) at 747 cm^{-1}.

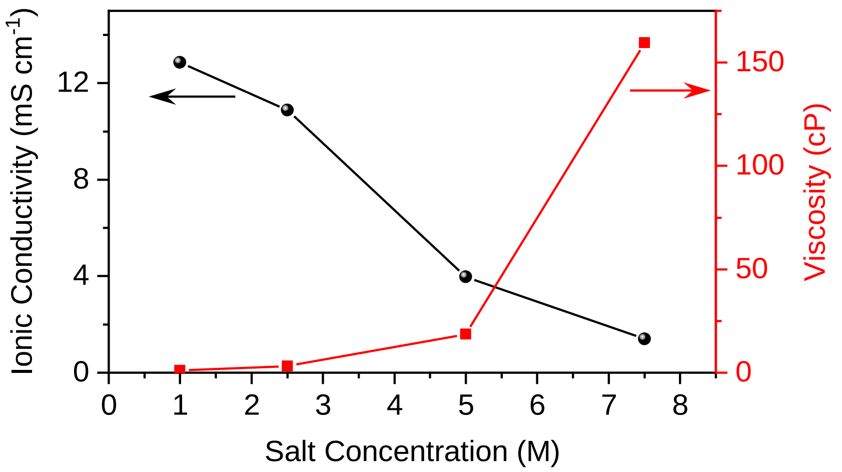

Figure S15. Ionic conductivity and viscosity of DOL-DME based electrolytes with increasing salt concentration, measured at 25°C. The decrease in the ionic conductivity and exponential increase in the viscosity of the electrolytes are consistent with previous reports.

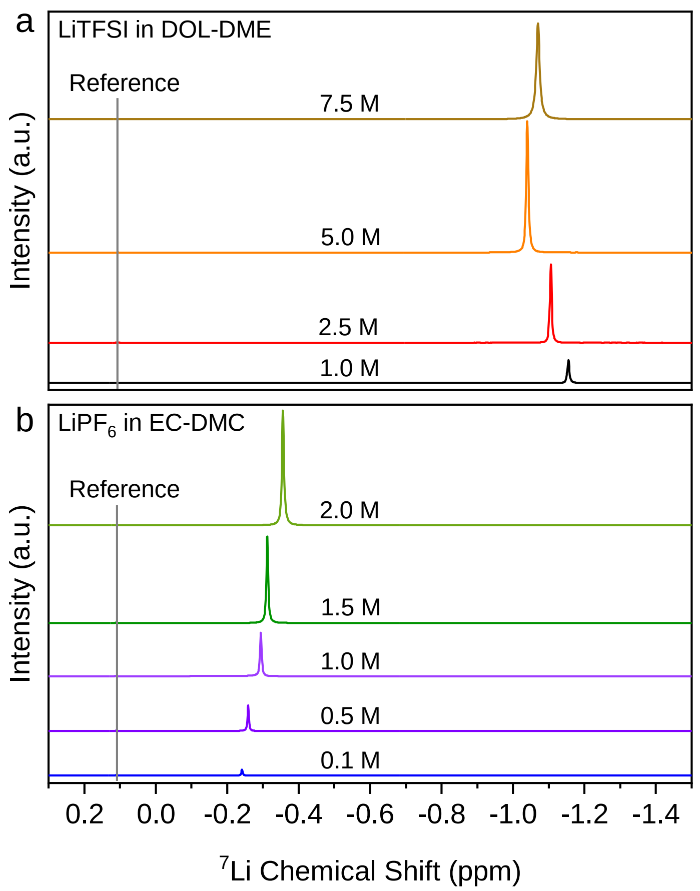

Figure S16. Changes of Li^+ environment in the solvation sheaths. ^{7}Li NMR spectra of the electrolytes incorporating (a) LiFTSI in DOL-DME and (b) $LiPF_6$ in EC-DMC with different salt concentrations.

The result shows that Li^+ cations in DOL-DME electrolytes are more de-shielded (weaker Li^+ solvation) upon increasing salt concentration, while it is vice versa in EC-DME electrolytes due to different electrolyte chemistry. The discrepancy makes us difficult to understand Li^+ solvation and transfer mechanism in different electrolyte systems. In contrast, the trend of stronger solvent and anions pairs is consistent in both EC-DMC (**Figure 3**a, b) and DOL-DME (**Figure 4**a, b) electrolyte systems, which are followed by the improved battery performance (**Figure 3**g, h and **Figure 4**c, d). The results confirm that the solvent and anion pairs are important interactions to study and probe the relationship between electrolyte microstructure and battery performance over a broad range of electrolyte compositions.

In addition to the discrepant behavior of Li^+ environment in the DOL-DME and EC-DMC electrolytes, in the superconcentrated electrolyte of 7.5 M DOL-DME, the Li^+ cations become shielded again (**Figure S16**a), while the battery performances are continuously enhanced (**Figure 3**g, h), this phenomenon definitely confuses researchers who believe in the conventional theory of first solvation shell of Li^+. Based on our findings, the possible reason for the reversely shielded Li^+ in the superconcentrated electrolyte is that the conjugated solvation network in the superconcentrated electrolyte diminishes the free $TFSI^-$ ion (SSIP) and promotes a significant population of contact ion pair (CIP) between $Li^+$and $TFSI^-$ (**Figure S14**b), which leads to more shielded Li^+. This reason is also supported by significant changes in the solvent-anion pairs from 5.0 M to 7.5 M (**Figure 3**a) as well as by the dramatic increase in the viscosity (**Figure S15**).

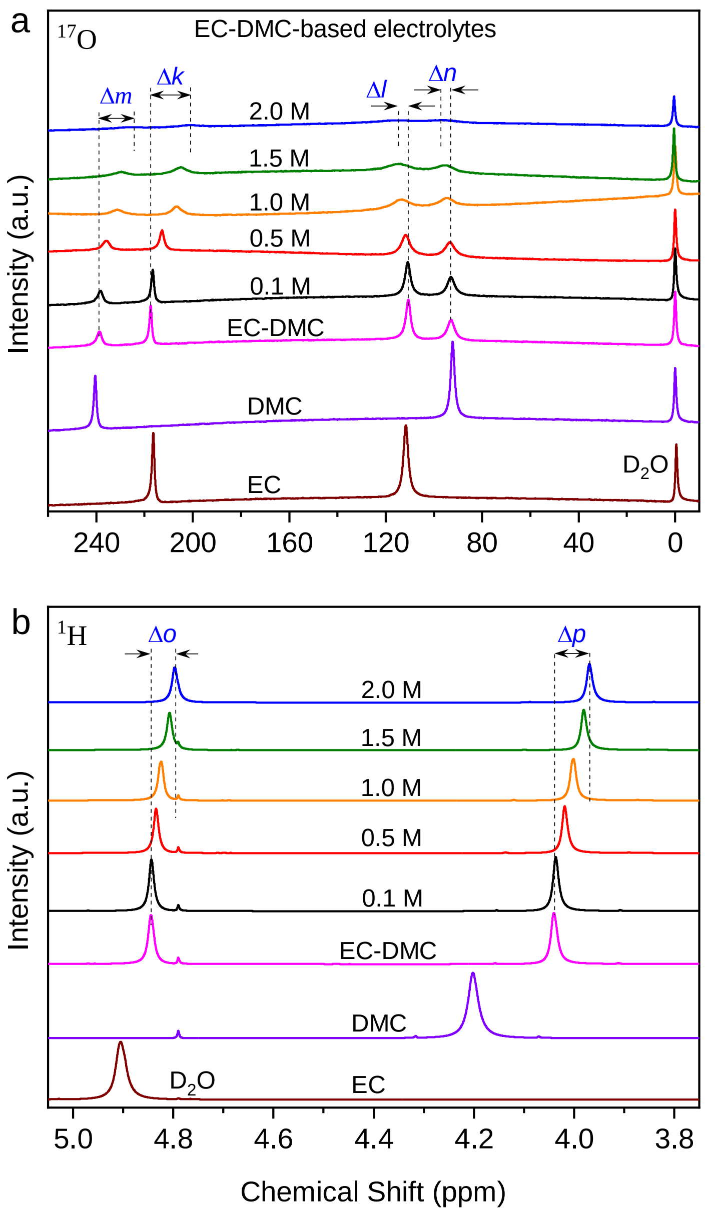

Figure S17. Investigation of carbonate-based electrolytes. (a) ^{17}O and (b) ^{1}H NMR spectra of electrolytes incorporating $LiPF_6$ in EC-DMC, suggesting significant changes of solvent environment in the electrolytes as compared to the neat EC-DMC solvents.

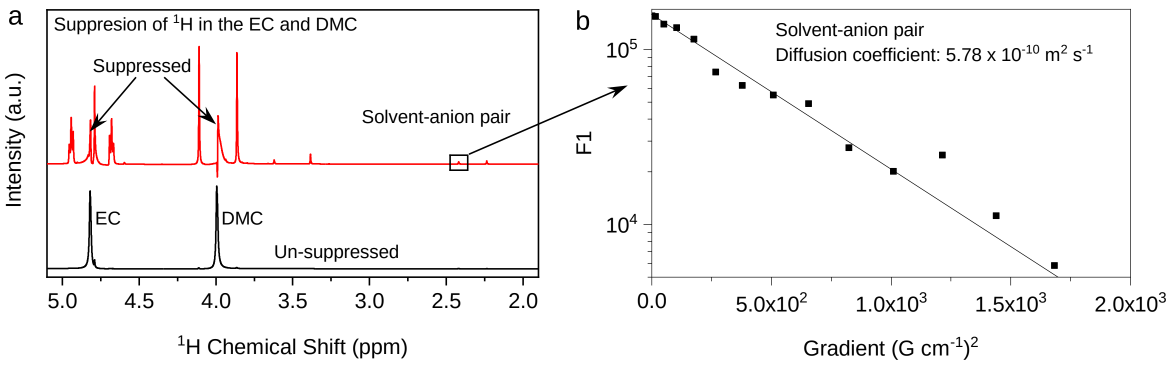

Figure S18. Diffusion coefficient of the solvent-anion pair. (a) Suppression of ^{1}H signal of EC and DMC solvents is performed to avoid interference in the solvent-anion pair signal caused by the dominant population of EC and DMC solvents. (b) Diffusion coefficient of the solvent-anion pair was measured with the suppressed EC and DMC signals, recorded from 1.0 M $LiPF_6$ EC-DMC electrolyte that shows the diffusion coefficient of 5.78 x 10^{-10} m^2 s^{-1}.

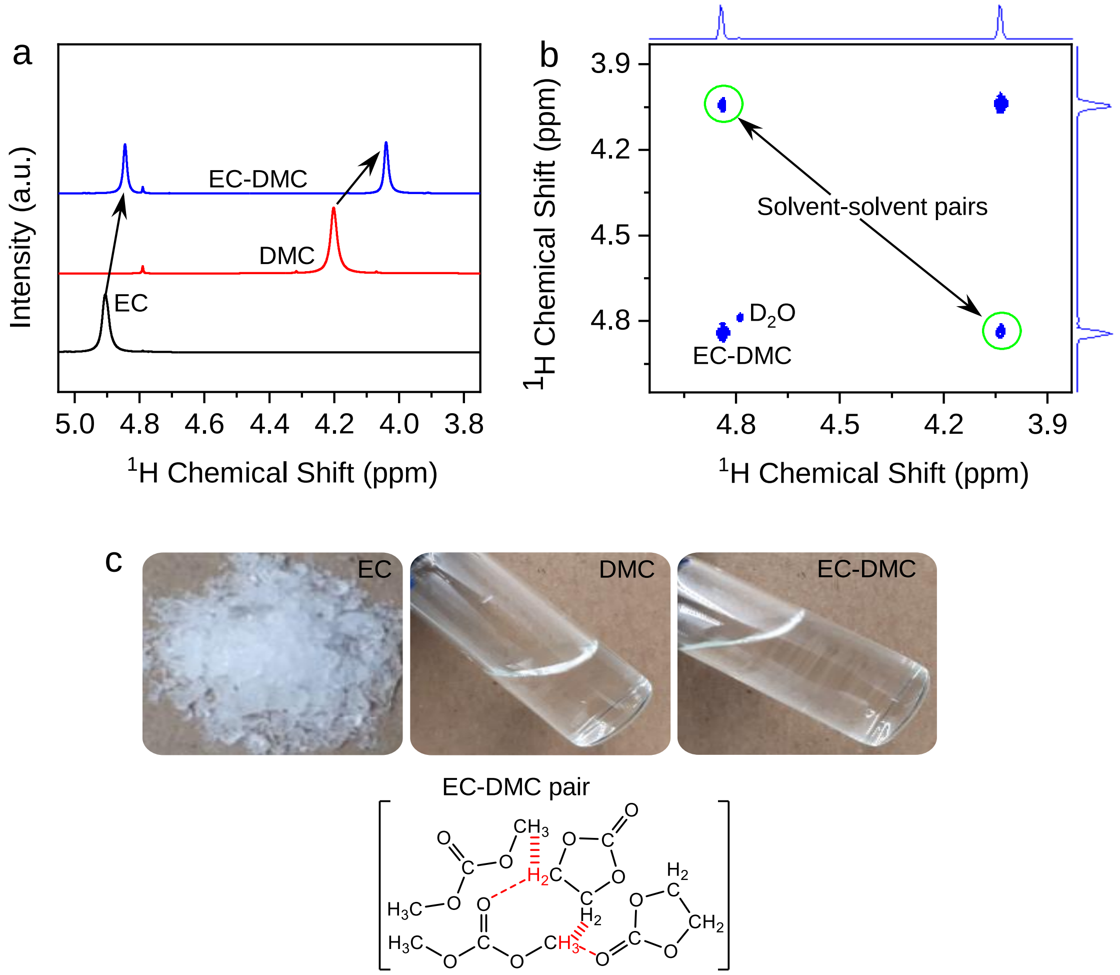

Figure S19. Solvent-solvent pair in the neat EC-DMC mixture. (a) ^{1}H NMR spectra of solvents showing their shift in the EC-DMC mixture. (b) COSY spectra showing solvent-solvent pairs in the neat EC-DMC mixture. (c) Photographs of the solid EC, liquid DMC, and a mixture of the two, with the plausible hydrogen bonding among the neat EC and DMC molecules.

Supporting References

(1) Valiev, M.; Bylaska, E. J.; Govind, N.; Kowalski, K.; Straatsma, T. P.; Van Dam, H. J. J.; Wang, D.; Nieplocha, J.; Apra, E.; Windus, T. L.; de Jong, W. A., NWChem: A comprehensive and scalable open-source solution for large scale molecular simulations. *Comput. Phys. Commun.* **2010**, *181* (9), 1477-1489.

(2) Becke, A. D., Density-functional thermochemistry. III. The role of exact exchange. *J. Chem. Phys.* **1993**, *98* (7), 5648-5652.

(3) Stephens, P. J.; Devlin, F. J.; Chabalowski, C. F.; Frisch, M. J., AB initio calculation of vibrational absorption and circular dichroism spectra using density functional force fields. *J. Phys. Chem.* **1994**, *98* (45), 11623-11627.

(4) Godbout, N.; Salahub, D. R.; Andzelm, J.; Wimmer, E., Optimization of Gaussian-type basis sets for local spin density functional calculations. Part I. Boron through neon, optimization technique and validation. *Can. J. Chem.* **1992**, *70* (2), 560-571.

(5) Grimme, S.; Antony, J.; Ehrlich, S.; Krieg, H., A consistent and accurate AB initio parametrization of density functional dispersion correction (DFT-D) for the 94 elements H-Pu. *J. Chem. Phys.* **2010**, *132* (15), 154104.
